# Molecular Details of a Coupled Binding and Folding Reaction between the Amyloid Precursor Protein and a Folded Domain

**DOI:** 10.1021/acschembio.1c00176

**Published:** 2021-06-23

**Authors:** Thomas M. T. Jensen, Christian R. O. Bartling, O. Andreas Karlsson, Emma Åberg, Linda M. Haugaard-Kedström, Kristian Strømgaard, Per Jemth

**Affiliations:** † Center for Biopharmaceuticals, Department of Drug Design and Pharmacology, University of Copenhagen, Jagtvej 162, 2100 Copenhagen, Denmark; ‡ Department of Medical Biochemistry and Microbiology, Uppsala University, BMC, Box 582, SE-75123 Uppsala, Sweden

## Abstract

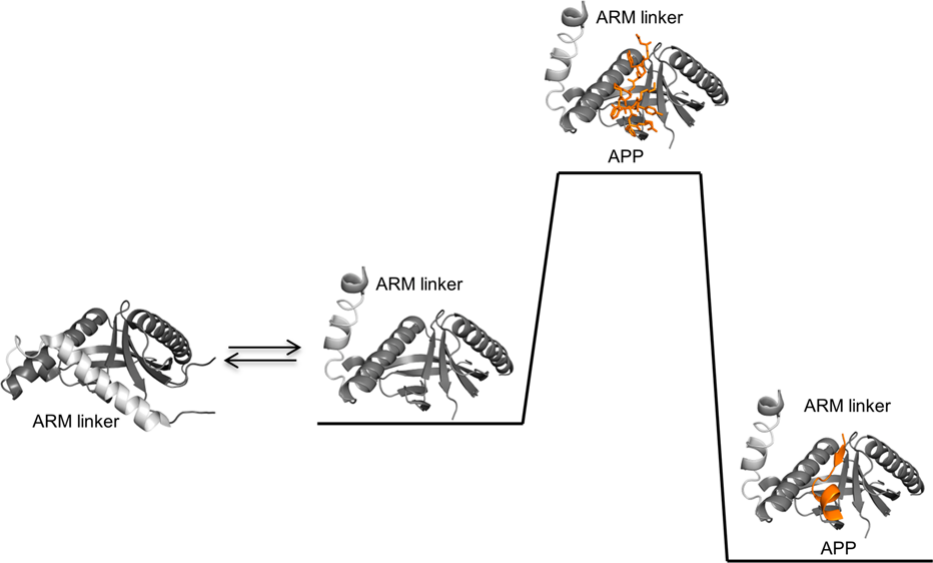

Intrinsically disordered regions in proteins often function as binding motifs in protein–protein interactions. The mechanistic aspects and molecular details of such coupled binding and folding reactions, which involve formation of multiple noncovalent bonds, have been broadly studied theoretically, but experimental data are scarce. Here, using a combination of protein semisynthesis to incorporate phosphorylated amino acids, backbone amide-to-ester modifications, side chain substitutions, and binding kinetics, we examined the interaction between the intrinsically disordered motif of amyloid precursor protein (APP) and the phosphotyrosine binding (PTB) domain of Mint2. We show that the interaction is regulated by a self-inhibitory segment of the PTB domain previously termed ARM. The helical ARM linker decreases the association rate constant 30-fold through a fast pre-equilibrium between an open and a closed state. Extensive side chain substitutions combined with kinetic experiments demonstrate that the rate-limiting transition state for the binding reaction is governed by native and non-native hydrophobic interactions and hydrogen bonds. Hydrophobic interactions were found to be particularly important during crossing of the transition state barrier. Furthermore, linear free energy relationships show that the overall coupled binding and folding reaction involves cooperative formation of interactions with roughly 30% native contacts formed at the transition state. Our data support an emerging picture of coupled binding and folding reactions following overall chemical principles similar to those of folding of globular protein domains but with greater malleability of ground and transition states.

## Introduction

Cellular regulation is highly reliant on protein–protein interactions (PPIs).^1−3^ Often, such PPIs are mediated by a globular well-folded protein interaction domain, which binds to an intrinsically disordered region of an interacting protein.^4^ Upon binding to the folded domain, the intrinsically disordered region typically adopts an ordered extended conformation, such as an α-helix, a β-strand, a coil, or a combination of secondary structures.^5^ A large body of computational studies^6^ and far fewer experimental studies^7−9^ have addressed such coupled binding and folding reactions. One general conclusion is that the binding involves an initial encounter complex, which rearranges into the native complex.^6,10^ The intrinsically disordered protein (IDP) can be viewed as a large ensemble of interconverting structures of similar Gibbs free energies, many of which can bind and form the encounter complex. In this model, some conformations may be preferred over others, and depending on the concentration of the interacting proteins and their conformations, the rate (or flux) via different parallel binding pathways is modulated.^11^ For example, a helical conformation in an IDP may bind with a rate constant that is higher than those of disordered conformations, but the latter are present at much higher concentrations such that binding predominantly occurs via a disordered conformation. Moreover, recent studies have found evidence of templated folding, whereby the structure and dynamics of the interaction partner influence the coupled binding and folding pathway of IDPs.^12−15^

**Figure 1:**
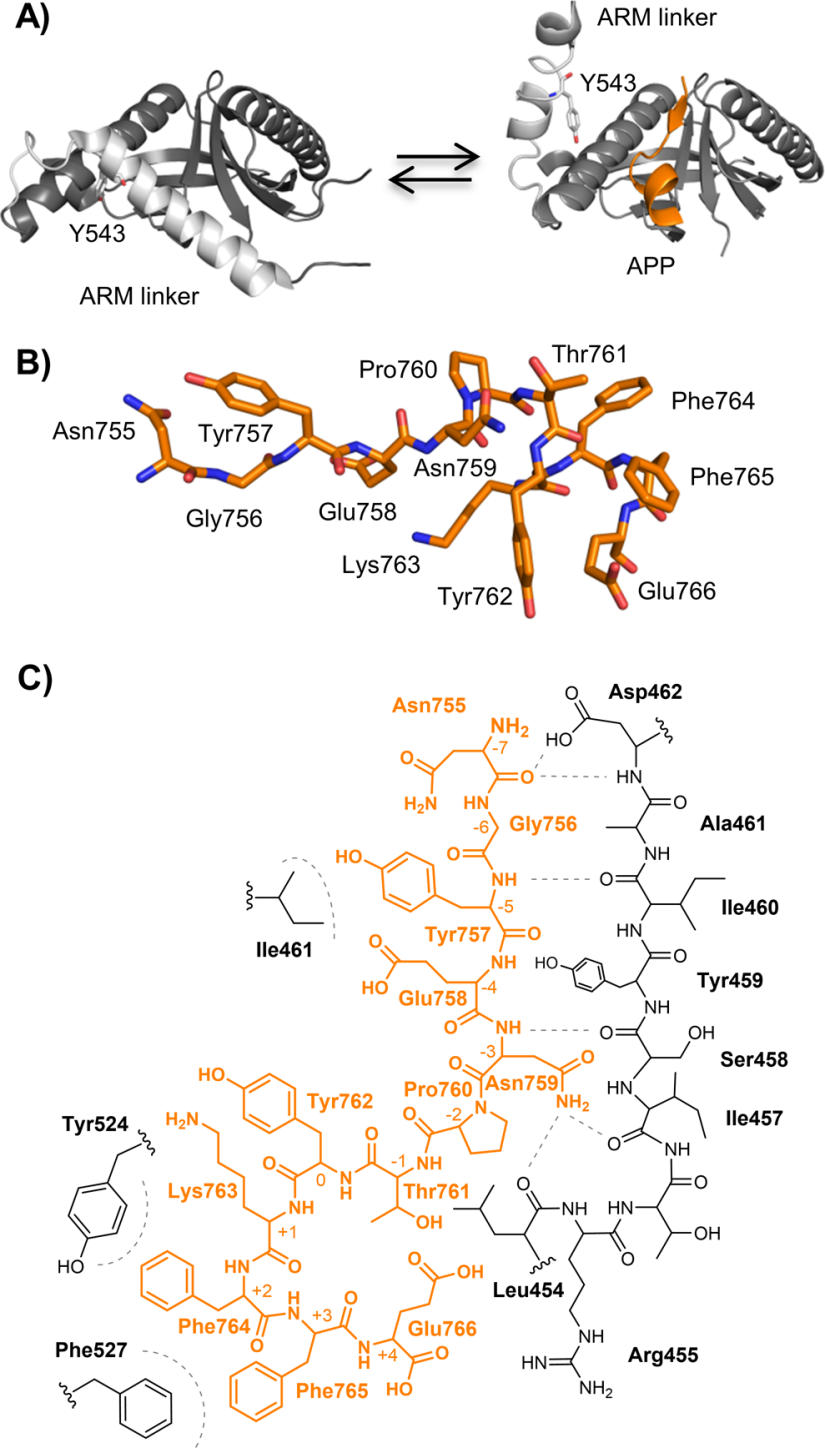
(A) Cartoon representation of the closed conformation of the PTB domain [left, Protein Data Bank (PDB) entry 3SUZ] and the open state (right, PDB entry 3SV1) co-crystallized with APP (orange). The proposed phosphorylation site, Y543, which affects the open–closed conformation of the ARM (light gray), is shown with sticks. (B) Stick representation of the APP (12-mer) peptide in its bound conformation (PDB entry 3sv1). (C) Lewis structure of APP (orange) binding to the PTB domain (black). Probed hydrophobic interactions and H-bonds are represented as gray dashed lines.

The intracellular C-terminus of amyloid precursor protein (APP) is intrinsically disordered, although a C-terminal α-helical structure is transiently present.^16,17^ Upon binding to the phosphotyrosine binding (PTB) domain of the munc-18 interaction protein (Mint), the APP C-terminus folds into a β-strand followed by a β-turn and a short α-helix (Figure 1).^18,19^ The APP–Mint interaction modulates the amyloidogenic processing pathway of APP, where β- and γ-secretase proteolytically cleave APP to form amyloid-β peptides leading to extracellular plaques characteristic of Alzheimer’s disease.^20−22^ It has been shown that the APP–Mint interaction is regulated by an open–closed conformation of a self-inhibitory α-helical linker (ARM) in the C-terminus of the Mint PTB domain (Figure 1A). In the closed state, the ARM linker occupies the APP binding site by forming extensive intramolecular hydrophobic interactions with the PTB domain. Phosphorylation of a tyrosine residue (Y543 in Mint2) in the ARM linker has been proposed to release the ARM autoinhibition.^23^

To understand this binding mechanism and to examine the proposed Mint2 regulation by phosphorylation of the ARM linker, we subjected the APP–PTB domain interaction to a detailed study combining protein engineering and kinetic studies. We found that the binding reaction is controlled by the ARM linker but that phosphorylation of the ARM does not modulate the binding. Intriguingly, the APP–PTB domain interaction displays properties distinct from those of previously investigated interactions involving IDPs. In particular, the change in the hydrophobicity of the side chains upon substitution correlates with the stability of the transition state more than with the stability of the native state, suggesting an unusually high degree of favorable non-native hydrophobic interactions in the transition state of this coupled binding and folding reaction.

## Methods

### General Peptide Synthesis

Unless otherwise stated, all starting materials were purchased from Sigma-Aldrich or Iris Biotech and used without further purification. Solid-phase peptide synthesis (SPPS) was performed on a 0.1 mmol scale on an automated Liberty Blue peptide synthesizer (CEM) using microwave-assisted couplings. Each coupling was achieved in dimethylformamide (DMF) with 4.0 equiv of Fmoc-AA-OH, 4.0 equiv of 2-(1-benzotriazol-1-yl)-1,1,3,3-tetramethyluronium hexafluorophosphate (HBTU), and 4.0 equiv of ethyl cyano(hydroxyimino)acetate (Oxyma Pure). Couplings were carried out at 75 °C for 3 min or for Asp-containing peptides at 50 °C for 5 min. Fmoc deprotection was achieved with either 20% piperidine in DMF or for Asp-containing peptides with a 9:1 mixture of methylpyrrolidone (NMP) and EtOH (5% piperazine and 0.1 M Oxyma Pure) at 75 °C for 3.5 min. Peptides were cleaved from the resin using a cleavage cocktail of trifluoroacetic acid (TFA), H_2_O, and triisopropylsilane (TIPS) (95:2.5:2.5) for 2 h at room temperature (RT) or for Cys-containing peptides TFA, H_2_O, TIPS, thioanisole, and ethanedithiol (EDT) (88:3:3:3:3) for 2 h at RT. Cleavage was followed by removal of TFA and ether precipitation. The precipitate was dissolved in 50% aqueous acetonitrile (MeCN) containing 0.1% TFA and subsequently lyophilized. The A-to-E modified peptides were synthesized as previously described.^22^

N-terminal carboxytetramethylrhodamine (TAMRA) was coupled on resin for 16 h at RT in a 1.5:1.5:3 5(6)-TAMRA (Anaspec Inc.)/(benzotriazol-1-yloxy)tripyrrolidinophosphonium hexafluorophosphate (PyBOP)/diisopropylethylamine (DIPEA) mixture dissolved in NMP.^24^ N-terminal dansyl was coupled at RT for 1 h using a 1:4:6 resin/dansyl chloride/DIPEA mixture dissolved in dichloromethane (DCM).

Phosphorylated peptides were obtained by coupling 2.0 equiv of Fmoc-Tyr[PO(NMe_2_)_2_]-OH followed by standard automated SPPS. Subsequently, cleavage was carried out using the cleavage cocktails described above with 10% H_2_O and cleavage for 6 h at RT.

Peptides were purified on a preparative reverse-phase high-performance liquid chromatography (RP-HPLC) system (Waters) with a C18 column (Zorbax, 300 SB-C18, 21.2 mm × 250 mm), using a binary buffer system of H_2_O, MeCN, and TFA (A, 95:5:0.1; B, 5:95:0.1). The collected fractions were characterized by electrospray ionization liquid (ESI) chromatography mass spectrometry (LC-MS) coupled to an Agilent 6410 triple quadrupole with a C18 column (Zorbax Eclipse XBD-C18, 4.6 mm × 50 mm) using a binary buffer system consisting of H_2_O, MeCN, and formic acid (A, 95:5:0.1; B, 5:95:0.1). The purity was analyzed at 214 nm on an analytical reverse-phase ultraperformance liquid chromatography (RP-UPLC) (Waters Acquity system) system with a C18 column (Acquity UPLC BEH C18 1.7 μm, 2.1 mm × 50 mm) using a binary buffer system consisting of H_2_O, MeCN, and TFA (A, 95:5:0.1; B, 5:95:0.1). The final peptide products were lyophilized (Table S1).

### Protein Expression and Purification

The human Mint2 DNA was purchased from Life Technologies and subcloned into a pRSET vector using primers 5′-CGGCGGCTCGAGCATCGAGGGTCGCAAAGAACTGCAGCTGG-3′ and 5′-CGGCGGGGATCCTCATCAGGTGGTCACCGGCG-3′ for PARM 364–570, 5′-CGGCGGGGATCCTCATCACAGATCTTCCGG-3′ for PTB 364–538, and 5′-CGGCGGGGATCCTCATCAAAAGTGGATCAGATCATCG-3′ for PARM 364–560. The PARM 364–532 thioester was generated as previously described^22^ using 5′-GTTTAACTTTAAGAAGGAGATATACATATGGAAGATCTGATTGATGGTATTATCTTTGC-3′ and 5′-GCAACTAATGCATCACCGGTAATACAACCATTGGCACGCAGAAATTC-3′. Site specific mutations were introduced by standard site-directed mutagenesis (Quickchange kit, Stratagene), and all DNA constructs were verified by DNA sequencing (GATC Biotech).

The plasmids containing the desired protein constructs were transformed into *Escherichia coli* BL21(DE3)pLysS (Invitrogen) and grown on Luria broth (LB)-agar plates containing ampicillin (100 μg mL^–1^) at 37 °C overnight. A few colonies were transferred into 100 mL of LB-medium including ampicillin (100 μg mL^–1^) and incubated for 16 h at 30 °C. Subsequently, the cells were transferred to either 0.5 or 1 L of LB medium containing ampicillin (100 μg mL^–1^) to give an OD_600_ of 0.1 and incubated at 37 °C. At an OD_600_ of 0.4–0.6, the expression cultures were induced with 0.1 mM isopropyl β-d-thiogalactoside (IPTG) at 37 °C for 4 h. After being induced, cells were harvested by centrifugation (10000 rcf, 10 min, 4 °C).

The His-tagged protein constructs were purified by suspending the pellets in lysis buffer [50 mM NaP_i_, 10 mM MgCl_2_, 25 μg/mL DNase, and cOmplete protease inhibitor tablets (1 tablet/50 mL) (Roche) (pH 7.4)] and disrupted by passing the lysate through a cell disruptor system (Constant System Ltd.) at 26000 psi. Subsequently, cell debris was removed by centrifugation (30000 rcf, 30 min, 4 °C), and the supernatant was loaded using a peristaltic pump onto 5 mL His Trap columns (GE Healthcare Life Science) and purified according to the standard protocol using wash buffer [20 mM imidazole, 150 mM NaCl, and 25 mM HEPES (pH 7.4)] and elution buffer [250 mM imidazole, 500 mM NaCl, and 25 mM HEPES (pH 7.4)]. Then 100 mM imidazole was used in wash buffer for PTB purification. The eluted protein was buffer exchanged into storage buffer [500 mM NaCl and 25 mM HEPES (pH 7.4)] using a Hiload 16/600 Superdex 75 pg column (GE Healthcare). All proteins were characterized by LC-MS (Agilent) with a C8 column (Poroshell, 300SB-C18, 2.1 mm × 75 mm), and the purity was determined by RP-UPLC (Waters Acquity system) with a C8 column (Acquity UPLC BEH C8 1.7 μm, 2.1 mm × 50 mm) (Table S2).

The N-terminal PARM 364–532 thioester was purified as described above until elution from the HisTrap column, where the protein was dialyzed into intein cleaving buffer [25 mM HEPES, 500 mM NaCl, and 2 M urea (pH 7.0)] using SnakeTubes (Sigma-Aldrich, 10000 Da molecular weight cutoff). Then 2-mercaptoethanesulfonic acid sodium salt (MesNa) was added to reach a final concentration of 200 mM, and thiolysis was allowed for 48 h at RT and pH 7.0. Subsequently, the pH was increased to 7.4 and the sample was loaded onto His-Trap columns and eluted with wash buffer to retain the GyrA_mini_ intein and uncleaved protein. The eluted MesNa thioester protein was then precipitated by dialyzing into water. Next, the precipitate was washed and lyophilized and characterized by LC-MS (Agilent) and UPLC (Waters) (Table S2).

### Semisynthesis

**Figure 2:**
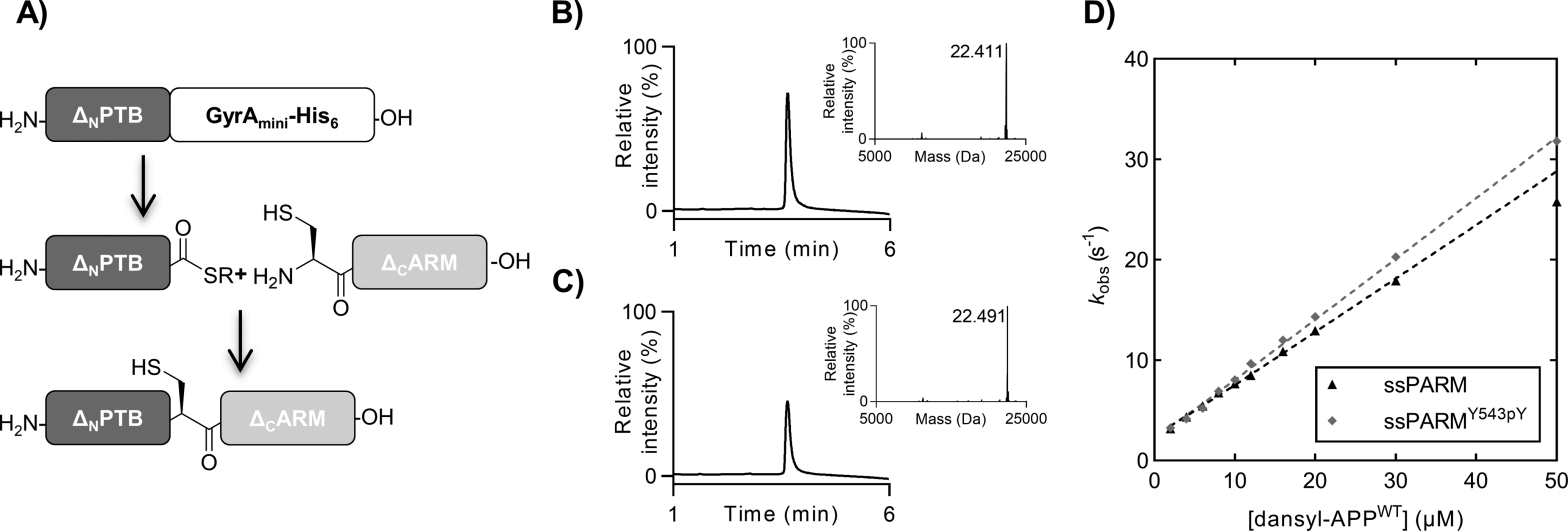
(A) PARM 364–532 (Δ_N_PARM) fused to a GyrA_mini_ intein was expressed and converted to MesNa thioester by intein thiolysis. Next, Δ_N_PARM was ligated with the synthetic ARM fragment containing the Y543pY substitution and subsequently refolded to yield the semisynthetic (ss) PARM domains. (B) UPLC–UV_214_ trace of ssPARM^WT^. The inset shows the LC-MS deconvoluted mass (expected mass of 22414). (C) UPLC–UV_214 nm_ trace of ssPARM^Y543pY^. The inset shows the LC-MS deconvoluted mass (expected mass of 22,494). (D) *k*_obs_ plotted vs the varying concentration of dansyl-APP for binding to ssPARM and ssPARM^Y543pY^.

The semisynthetic constructs were prepared by dissolving the Cys peptide and PARM-MesNa protein in ligation buffer [6 M GuHCl, 200 mM NaP_i_, 100 mM mercaptophenyl acetic acid (MPAA), and 17 mM TCEP (pH 7.0)]. After ligations for 4 h, the reaction was complete as measured by LC-MS and the semisynthetic protein was refolded and purified into storage buffer using a Hiload 16/600 Superdex 75 pg column (GE Healthcare). The semisynthetic proteins were characterized by RP-UPLC at 214 nm, 16% sodium dodecyl sulfate–polyacrylamide gel electrophoresis, and LC-MS. PARM A-to-E variants were generated as described previously and characterized by LC-MS and RP-UPLC at 214 nm (Figure 2 and Table S2).^22^

### Circular Dichroism

Circular dichroism experiments were performed on a Jasco J-1500 (Jasco Inc.) spectrometer in a 1 mm quartz cuvette using a protein concentration of 15 μM in 500 mM NaCl and 25 mM HEPES (pH 7.4). Data were obtained in millidegree ellipticity (*m*_0_) and converted to mean residue ellipticity (Θ_MRE_) using the equation Θ_MRE_ = *m*_0_(L[protein]*n*)^−1^, where [protein] is the concentration of protein in molar, *n* is the number of backbone amides in the protein, and *L* is the path length of the cuvette in millimeters.

### Fluorescence Polarization

Binding affinities were determined in a 384-well plate format (Corning Life Science) using a Safire^2^ plate reader (Tecan) using (TAMRA)-NNG-NGYENPTYKFFE as a probe (excitation at 530 nm and emission at 580 nm) at 50 nM. The *G*-factor was calibrated to give an initial millipolarization of 20, and the instrumental *Z*-factor was adjusted to the maximum fluorescence. All measurements were conducted in 500 mM NaCl, 25 mM HEPES, and 1% BSA (pH 7.4) at 25 °C. To determine the affinity of the protein construct, a saturation assay was conducted in which the polarization was plotted as a function of the protein concentration and fitted to a one-site binding model to yield the *K*_d_ at 50% saturation. To measure affinities between dansylated peptides, a competition assay with fixed protein (2–30 μM) and probe concentrations (50 nM) and varying peptide concentrations from 0.1 to 1000 μM was conducted. The experiments were performed in technical triplicate, and the data were fitted to a sigmoidal dose–response curve. The *K*_i_ value was calculated according to the method of Nikolovska-Coleska et al.^25^

### Kinetics

Kinetic binding rate constants were obtained using an upgraded SX17 stopped flow spectrometer (Applied Photophysics), and all experiments were conducted at 25 °C in 500 mM NaCl and 25 mM HEPES (pH 7.4). The relatively high NaCl concentration was used to improve the solubility of the proteins. The change in tryptophan fluorescence was measured using a band-pass filter (excitation at 280 nm and emission at 330 ± 30 nm) when mixing varying concentrations (2–50 μM) of the dansyl peptides with 1 μM protein or 0.5 μM semisynthetic proteins. Kinetic transients were fitted to a single-exponential equation (PARM variants) or a double exponential equation (PTB variants) to obtain the observed rate constant (*k*_obs_). For the PARM variants, there was a small deviation from perfect single-exponential behavior, but the difference in *k*_obs_ values obtained from fitting a double-exponential equation was too small (3-fold) for a quantitative analysis. For PTB variants, we used the *k*_obs_ value from the fast kinetic phase for the analysis. Reassuringly, for both PARM and PTB variants, the *K*_d_ values calculated from *k*_off_/*k*_on_ (assuming apparent one-state) correlated well with *K*_d_ from both calorimetric and fluorescence polarization experiments, and we are therefore confident about our analysis. Each kinetic transient used to determine *k*_obs_ was an average of three or more individual technical replicates measured back to back. *k*_obs_ values were plotted as a function of peptide concentration and fitted to the equation *k*_obs_ = [*k*_on_^2^([protein]_0_ – [peptide]_0_)^2^ + *k*_off_^2^ + 2*k*_on_*k*_off_([protein] – [peptide]_0_)]^0.5^, where [protein]_0_ and [peptide]_0_ are the total concentrations of protein and dansylated peptide, respectively, in micromolar. We report the errors in *k*_on_ and *k*_off_ from the curve fitting. The error in *k*_on_ from three independent replicates in these types of experiments is usually close to the fitting error. The error in the extrapolated *k*_off_ is estimated to be ∼10%. If *k*_off_ was not well-defined by fitting from the equation presented above (usually when it is <10 s^–1^), a displacement experiment was performed; here a complex of 1 μM dansyl peptide and 1 μM protein was mixed with 50–150 μM APP^WT^ (NGYENPTYKFFE) as previously described using a cutoff filter (excitation at 330 nm and emission at >420 nm).^26^ At high APP^WT^ concentrations, the *k*_obs_ value approaches *k*_off_. For these *k*_off_ determinations, we report the standard error of the mean of three replicates. Φ values were defined as ΔΔ*G*_TS_/ΔΔ*G*_eq_, where ΔΔ*G*_TS_ = *RT* ln(*k*_on_^WT^/*k*_on_^mut.^) and ΔΔ*G*_eq_ = *RT* ln(*K*_d_^mut.^/*K*_eq_^WT^). Calculated coupling free energies were defined as ΔΔΔ*G*_Eq_ = *RT* ln(*K*_d_^mut.:WT^*K*_d_^WT:mut.^/*K*_d_^WT:WT^*K*_d_^mut.:mut.^) or ΔΔΔ*G*_TS_ = *RT* ln(*k*_on_^WT:WT^*k*_on_^mut.:mut.^/*k*_on_^mut.:WT^*k*_on_^WT:mut.^) as previously described.^27^ Propagated errors are reported. In general, errors in Φ values in the tables in the Supporting Information are therefore underestimated, and the true error is usually on the order of ±0.1. Similarly, true errors in the Gibbs free energy may be underestimated and are at least ±0.1 kcal mol^–1^.

### Isothermal Titration Calorimetry

Isothermal titration calorimetry experiments were conducted on a MicroCal iTC200 System (Malvern Instruments). The proteins were dialyzed against a buffer containing 500 mM NaCl and 25 mM HEPES (pH 7.4). Protein concentrations were determined using calculated extinction coefficients at 280 nm and diluted to the experimental concentration using dialysis buffer. The APP peptide was placed in the syringe with a 10-fold higher concentration compared to that of PTB/PARM in the sample cell. During injections, the sample cell was held at 25 °C. The data were analyzed with ITC Origin software and fitted to a one-binding site model. The binding stoichiometry was determined to be between 0.9 and 1.1.

### Data Analysis

Figures of structural models were prepared with PyMOL (DeLano Scientific). Analysis of data was performed in excel 2010 (Microsoft) and GraphPad Prism 7.00 (GraphPad Software Inc.).

## Results

### Design of Pseudo-Wild Type and Mutant Variants of the PTB Domain and APP

To examine the role and mechanism of phosphorylation in the binding reaction between APP and the PTB domain of Mint2, we subjected the Mint2 PTB domain and APP variants to side chain and backbone modifications and kinetic experiments. Because the Mint2 PTB domain does not contain a suitable probe for fluorescence detection in stopped flow measurements, a Trp residue (Y524W) was introduced into all PTB domain variants. This pseudo-wild type (pWT) PARM domain (residues 364–570, i.e., PTB and the ARM linker) containing the Y524W mutation displayed an APP binding affinity and a secondary structure similar to those of PARM^WT^ (Figures S1 and S2). Furthermore, the kinetic association (*k*_on_) and dissociation rate constants (*k*_off_) were similar for the binding of APP to pWT PARM (*k*_on_ = 0.39 ± 0.004 μM^–1^ s^–1^, and *k*_off_ = 2.3 ± 0.08 s^–1^) compared to those for the binding of APP to PARM^WT^ (*k*_on_ = 0.38 ± 0.003 μM^–1^ s^–1^, and *k*_off_ = 1.2 ± 0.05 s^–1^). N-Terminal dansyl labeling of the APP^WT^ peptide to increase the change in the fluorescence intensity upon binding also did not affect the binding kinetics (Figure S1).

The X-ray crystal structure of the apo version of the PTB domain shows that the ARM linker covers the APP binding site (Figure 1A). We therefore introduced mutations into PARM to destabilize the interaction between the ARM linker and the PTB domain or remove it completely to compare the binding properties of the PTB domain in the open and closed conformations. Hence, we expressed and purified pWT PARM, PARM^Y543A^, PARM^Y543E^, and PTB without the ARM linker (residues 364–538) (Figures S2 and S3 and Table S2). Residue 543 (Y543) was selected to introduce point mutations because Y543 was previously proposed to regulate the binding of APP (Figure 1A).^23^ In addition to the Y543E phosphomimetic substitution, we introduced a phosphorylated tyrosine (Y543pY) by protein semisynthesis. The substitutions in APP were selected to probe the secondary structure of APP (P760A/G, K763A/G, F764A/G, and F765A/G) or to evaluate key residues in the binding interface (Y757F, Y762F, F764Nal1, and F765Nal1) (Figure 1B,C and Table S1).^22^ Notably, tyrosine phosphorylation of peptide ligands is important for the binding to other PTB domains but is not believed to be relevant for the APP–Mint2 interaction.^18,28,29^ To test this, we also introduced a phosphotyrosine in APP (Y762pY). In addition, we wanted to probe the backbone–hydrogen bond network between strand β5 of the PTB domain and APP. Therefore, we generated a range of amide to ester (A-to-E) modified APP (G756γ, Y757ψ, E758ε, N759ν, and F765φ) peptides and PARM (Y459ψ, I460ι, A461α, and D462δ) domains (Figure 1C), and the latter was achieved by protein semisynthesis as described previously (Tables S1 and S2).^22^

### The Binding Kinetics Follow an Apparent Two-State Mechanism

**Figure 3:**
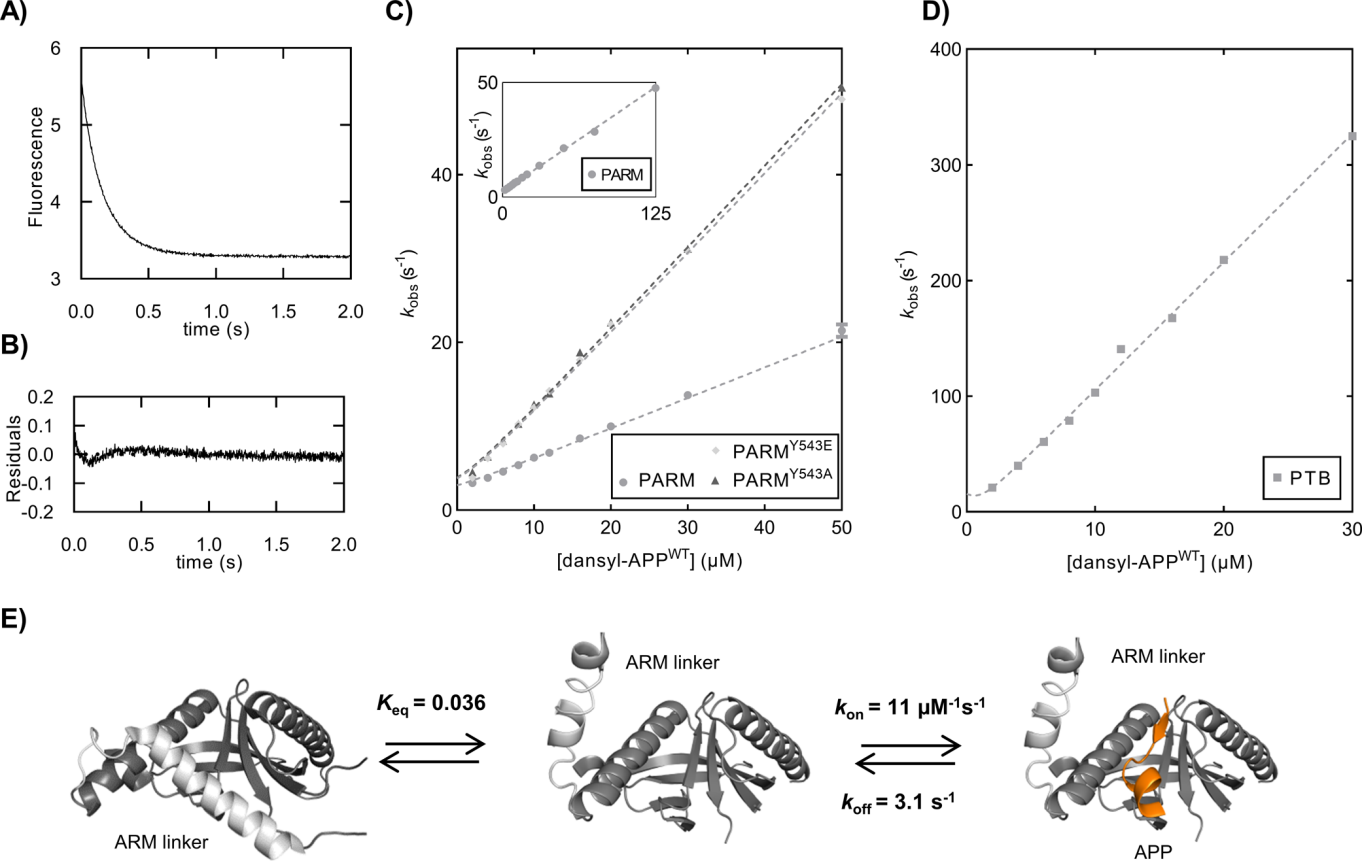
(A) Example of a kinetic binding trace between dansyl-APP (10 μM) and PARM (1 μM). (B) Residuals of a single-exponential fit. (C and D) Observed rate constant (*k*_obs_) plotted vs varying concentrations of dansyl-APP for the binding to PARM variants. The inset in panel C shows the binding between dansyl-APP and PARM at an extended concentration range of dansyl-APP showing that *k*_obs_ increases linearly at least up to 50 s^–1^. The rate-limiting open–closed equilibrium must therefore occur on a time scale of ≪1/50 s. (E) Reaction scheme with estimated rate and equilibrium constants for the binding reaction between PARM and APP. A *K*_eq_ of 0.036 was calculated on the basis of the *k*_on_ values for PTB (11 μM^–1^ s^–1^) and PARM (0.39 μM^–1^ s^–1^), under the assumption that *k*_on_^PARM^ = *k*_on_^PTB^*K*_eq_/(1 + *K*_eq_) and that dissociation of the ARM linker in PARM provides similar access of APP to the binding pocket as to the PTB domain. The ratio of *K*_d_ values for PARM and PTB (0.057) is close to *K*_eq_, but not identical due to differences in *k*_off_.

Using stopped flow spectroscopy and monitoring the change in fluorescence upon binding, we measured the association and dissociation rate constants of all APP variants against the four PTB domain variants, yielding a total of 56 kinetic binding experiments (Figure S4), each consisting of 5–10 observed rate constants, *k*_obs_. The kinetic transients, used to determine *k*_obs_ values, were manifested as a decrease in fluorescence resulting from the binding. The transients displayed an excellent signal-to-noise ratio and were fitted to a single exponential (PARM variants) assuming a simple two-state binding (Figure 3A). The residuals from the fitting were not perfectly single exponential but displayed a trend, which potentially results from a more complex binding mechanism. However, on the basis of the small deviation of the residuals from random noise, in relation to the large amplitude of the kinetic trace, we deemed that a single exponential mirrored the data sufficiently well (Figure 3B). Indeed, analyses using a double-exponential function resulted in *k*_obs_ values that differed by a factor of only 3, precluding a meaningful interpretation. For PTB, there was a larger difference (∼10-fold) between the observed rate constants when analyzed by a double-exponential function. We could not interpret the slow phase mechanistically but speculate that it could originate from either heterogeneity in the PTB population, transient dimer formation, or a minor conformational transition, which is affecting the sensitive Trp fluorescence. The observed rate constant obtained from the single-exponential curve fitting was plotted against APP concentration to estimate values of *k*_on_ and *k*_off_. The *k*_off_ value was determined independently in a displacement experiment in those cases in which it was low and thus ambiguous from extrapolation to zero peptide concentration. The affinities calculated from stopped flow experiments (*k*_off_/*k*_on_) (Table S3) were in good agreement with the affinities determined in our fluorescence polarization (FP) binding assay (Figure S5 and Table S4) and by isothermal titration calorimetry (ITC) (Figure S6), corroborating the apparent two-state nature of the interaction and the validity of analyzing the stopped flow measurements using a single-exponential (PARM) or double-exponential (PTB) function. Analysis and interpretation of the kinetic data are described in the following sections.

### The ARM Domain Restricts Binding to the PTB Domain

The binding of APP^WT^ to the four PTB domain variants all resulted in a *k*_off_ of approximately 2 s^–1^, indicating that the ARM linker does not affect the dissociation of APP (Table S3). In contrast, deletion of the ARM linker resulted in a 30-fold increase in the *k*_on_ value (PARM vs PTB) (Figure 3C,D). The Y543A and Y543E mutations in the ARM linker resulted in a 2.5-fold increase in *k*_on_, hence only slightly releasing ARM–linker inhibition. This observation was not affected by substitutions in APP as all APP variants displayed a similar behavior for *k*_on_ and *k*_off_ (Figure S7), with an average increase in *k*_on_ of 3-fold (PARM^Y543A^ and PARM^Y543E^) and 31-fold (PTB), respectively, compared to PARM, and with similar *k*_off_ values for all PARM variants. These results suggest that the ARM linker restricts the binding of APP to the PTB domain via a fast pre-equilibrium between an open conformation and a closed conformation occurring on a time scale much faster than 20 ms, as shown by the linear dependence of *k*_obs_ up to 125 μM dansyl-APP^WT^ (Figure 3C, inset). A reaction scheme with approximate rate constants for the PARM–APP interaction is drawn in Figure 3E. The scheme is based on data obtained for PARM–APP and PTB–APP kinetic experiments and assumes “open access” for APP to the PTB domain, while the ARM linker must dissociate from the binding pocket to allow APP binding.

### APP Binding Is Not Regulated by Phosphorylation of Tyr543 in PARM

Next, we wanted to examine if phosphorylation of Tyr543 could regulate the open–closed conformation of the PARM domain. We introduced the site specific phosphorylation by protein semisynthesis.^30,31^ A two-segment semisynthetic strategy was chosen in which the tyrosine phosphorylation (pY) was introduced synthetically in the ARM segment and the PTB domain was expressed as a thioester using an intein approach as previously described (Figure 2A).^22^ We generated Δ_N_PTB-sodium 2-mercaptoethanesulfonate (MesNa) from thiolysis of expressed PTB 364–532 C-terminally fused to GyrA_mini_ and synthesized ΔARM^WT^ 533–560 and ΔARM^Y543pY^ 533–560 using SPPS (Tables S1 and S2).^32^ With all fragments in hand, we ligated and refolded the WT semisynthetic (ss) PARM^WT^ as a control and phosphorylated ssPARM^Y543pY^ to generate the desired proteins with good purity (Figure 2B,C and Figure S8). The secondary structure of the semisynthetic proteins was verified by CD, and FP experiments confirmed the binding of APP to ssPARM^WT^ and ssPARM^Y543pY^ (Figures S2 and S3). Unexpectedly, the binding of APP to ssPARM^Y543pY^ (*k*_on_ = 0.61 ± 0.008 μM^–1^ s^–1^, and *k*_off_ = 2.0 ± 0.01 s^–1^) resulted in similar binding kinetics as observed for ssPARM^WT^ (*k*_on_ = 0.53 ± 0.008 μM^–1^ s^–1^, and *k*_off_ = 2.0 ± 0.01 s^–1^) (Figure 2D). Thus, binding of the ARM linker to the Mint PTB domain does not seem to be regulated by phosphorylation of Tyr543 as previously proposed. Furthermore, our experiments illustrate the particular challenge of using Glu residues as phosphomimics to predict the effect of tyrosine phosphorylation as recently described for other proteins.^33^

### Linear Free Energy Relationships (LFERs) Show a Cooperative Formation of Interactions in the Binding Site

Having established that the ARM linker restricts APP binding, we further analyzed the binding kinetics of the APP side chain substitutions. Most APP substitutions led to a destabilization in both the transition state (calculated from association rate constants) and at equilibrium (calculated from *K*_d_). For example, the successive substitutions at positions 760 and 764, P760 → A → G and F764 → A → G, respectively, each destabilized the equilibrium by ∼1 kcal mol^–1^, indicating that both tertiary and secondary structures were perturbed by the substitutions. In addition, the Y762pY variant also led to a dramatic decrease in affinity, confirming that phosphorylation of APP weakens binding to the Mint2 PTB domain. On the contrary, two noncanonical naphthyl substitutions (F764Nal1 and F765Nal1) resulted in a stabilization of both the transition state and the bound state, suggesting a pronounced role of hydrophobic effects in the APP–Mint2 interaction.

**Figure 4:**
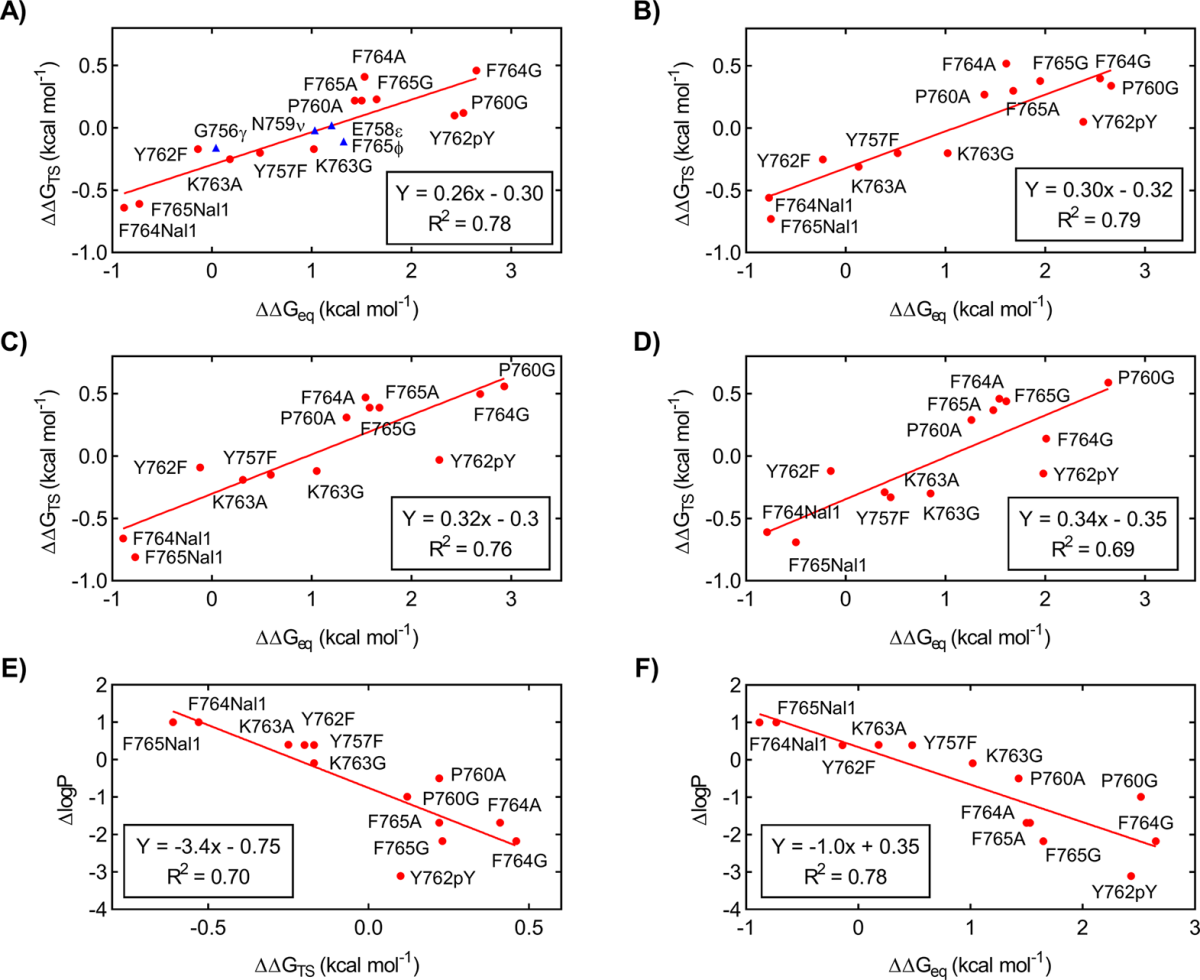
(A–D) Free energy difference in the transition state (ΔΔ*G*_TS_) plotted vs the free energy difference at equilibrium (ΔΔ*G*_eq_) of APP variants for binding to PARM, PARM^Y543A^, PARM^Y543E^, and PTB, respectively. The data were fitted to a straight line as indicated in the plot. ΔΔ*G* was defined as the free energy difference between the variant and wild type. A-to-E substitutions (triangles) are shown for comparison but not included in the fitting. (B and C) Change in hydrophobicity of APP substitutions plotted vs ΔΔ*G* for the binding of APP to PARM. The change in hydrophobicity from the APP variant to APP^WT^ (Δlog *P*) was plotted vs (B) ΔΔ*G*_TS_ or (C) ΔΔ*G*_eq_ for the interaction with PARM and fitted to a straight line. ΔLog *P* (*n*-octanol/water) was calculated using ChemBioDraw on the basis of Crippens fragmentation.^35^

We further analyzed the results using LFERs by calculating the change in free energy between the APP variants and APP^WT^ and plotting the change in free energy at the transition state (ΔΔ*G*_TS_) versus the change in free energy at equilibrium (ΔΔ*G*_eq_) (Figure 4A–D). The resulting LFERs were similar for all PTB domain variants with a slope of ∼0.3, demonstrating that the substitutions in APP affect the binding kinetics to a similar degree regardless of whether the ARM linker is present. This consistent behavior among the PTB domain variants shows that the ΔΔ*G* values calculated for APP variants reflect the rate-limiting transition state for complex formation and are not affected by the fast pre-equilibrium involving the ARM linker. Furthermore, the ΔΔ*G* values resulting from the A-to-E variants in APP fitted well to the same straight line as those from side chain substitutions in the LFERs (Figure 4A). By analogy with protein folding studies,^34^ this suggests that all noncovalent bonds of the interaction form cooperatively and with an average degree of formation of ∼30% at the top of the transition state barrier.

### The Change in Hydrophobicity Correlates with ΔΔ*G*

To elucidate if the increased affinity of the hydrophobic naphthyl substitution in APP (F764Nal and F765Nal1) was a general feature, we investigated the relationship between the change in hydrophobicity and ΔΔ*G* for binding upon substitution. We estimated the change in hydrophobicity from computational log *P* values (partition coefficient between *n*-octanol and water) for the respective amino acid side chain.^35^ The difference in log *P* for the amino acid substitutions (APP variant vs APP^WT^, Δlog *P*) was plotted versus either ΔΔ*G*_eq_ or ΔΔ*G*_TS_ for the binding of APP to PARM (Figure 4E,F). This revealed that the free energy difference correlates with the change in hydrophobicity of the amino acid side chains. This unusually strong correlation with hydrophobicity (slopes of −1.0 for ΔΔ*G*_eq_ and −3.4 for ΔΔ*G*_TS_) has not been reported for other IDP interactions previously characterized by kinetics and protein engineering (Figure S9).^36−40^

### Native and Non-native Interactions in the Rate-Limiting Transition State

**Figure 5:**
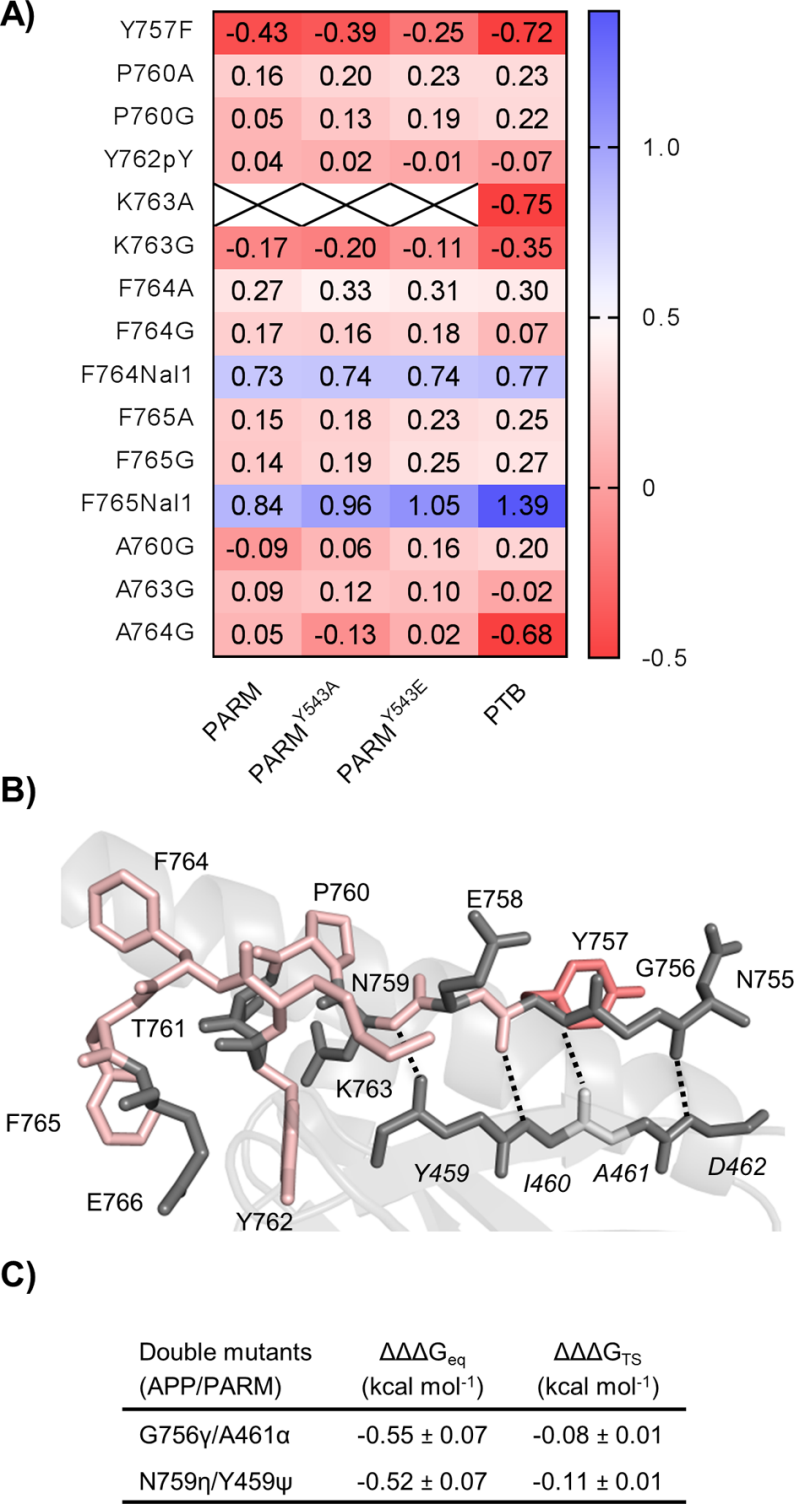
(A) Heat map showing the Φ values of APP variants toward the PTB domain variants. The Φ value is defined as ΔΔ*G*_TS_/ΔΔ*G*_eq_ and reported if ΔΔ*G*_eq_ is >0.35 or <−0.35 kcal mol^–1^. (B) Calculated Φ values of the probed APP residues were color-coded and mapped onto a stick representation of the bound conformation. The PTB domain is shown as a cartoon with strand β5 shown as sticks (Protein Data Bank entry 3SV1). The backbone Φ values of P760, K763, F764, and F765 were calculated from the difference between the glycine variant and the alanine variant. Φ values are colored blue for values of >0.6, white for values of 0.3–0.6, light red for values of −0.3 to 0.3, and red for values of <−0.3. (C) Calculated H-bond coupling free energies.

To map the transition state of the binding reaction in more detail, we calculated Φ values from the data set. In such an analysis, the degree of native interaction(s) formed by the substituted side chains in the transition state is estimated by taking the ratio of ΔΔ*G*_TS_ and ΔΔ*G*_eq_. Thus, if an interaction is fully formed in the transition state, the effect of the substitution in the transition state ΔΔ*G*_TS_ is expected to equal the effect in the bound state ΔΔ*G*_eq_, and Φ = 1. On the contrary, if the interaction has not begun to form in the transition state the ΔΔ*G*_TS_ value is zero and Φ = 0; thus, the probed interaction forms late in the binding reaction. Note that for an apparent two-state reaction as in the case presented here (not considering the fast open–closed pre-equilibrium), the Φ value can be calculated using either *k*_on_ or *k*_off_. The error in the Φ value will be large when ΔΔ*G*_eq_ approaches zero (i.e., no change in *K*_d_ upon substitution), and in the work presented here, we used a cutoff for calculating Φ values of ΔΔ*G*_eq_ > 0.35 kcal mol^–1^, which is based on the precision and accuracy of the measured rate constants (Table S5).^26^ As indicated from the LFERs, the Φ values for the PTB domain variants were similar and generally low, suggesting that the majority of the residues obtain their native conformation and interactions on the downhill side of the transition state barrier (Figure 5A,B). Some APP variants resulted in an increase in both *k*_on_ and *k*_off_ and thus a negative Φ value, for example Y757F and K763 → A → G. One interpretation of this is that the hydroxyl group of the Tyr and the backbone of Lys make unfavorable non-native contact(s) and conformation(s), respectively, in the transition state but favorable interactions in the bound state. However, negative Φ values are not straightforward to interpret,^41^ and another explanation would be ground state effects, i.e., that not only the bound state is destabilized by mutation but also the free state of APP. At positions 764 and 765, the native residues (Phe) partially form native interactions in the transition state (Φ = 0.07–0.25). Substituting the benzyl ring for a naphthyl at these positions increased the affinity of the complex with an increase in *k*_on_. Thus, these noncanonical substitutions resulted in Φ values of ∼1, suggesting that the naphthyl groups provide an alternative binding interface and form fully native (and favorable) interactions in the transition state (*k*_off_ values were similar to those of the wild type). Furthermore, the APP A-to-E substitution probing the β-strand (G756γ, E758ε, and N759ν) and the α-helix (F765φ) generally affected the *k*_off_, resulting in Φ values close to 0 (Table S6). No Φ value could be calculated for G756γ due to a ΔΔ*G*_eq_ of <0.35 kcal mol^–1^, and Y757ψ did not give a kinetic transient in the stopped flow experiment, most likely due to a *k*_obs_ outside the range of the instrument (>300 s^–1^) (Figure S10). In general, the Ala to Gly substitutions and F765φ probing the helical and β-turn formation resulted in Φ values close to 0, suggesting that this secondary structure is formed late in the binding interaction (Figure 5A,B).

### APP/PTB Backbone H-Bonds Are Energetically Unfavorable

To complement the APP A-to-E backbone substitutions and enable a double-mutant cycle analysis to quantify the backbone H-bond energy, we introduced four A-to-E substitutions (Y459ψ, I460ι, A461α, and D462δ) into strand β5 of the PTB domain. In brief, the A-to-E substitutions were introduced using our previously developed semisynthetic strategy to generate PARM-pWT (R455K, C483A, C501A, and C566A) A-to-E variants relying on a three-segment approach by inserting the Y524W mutation into the C-terminally expressed fragment. CD confirmed that all four PARM-pWT A-to-E variants had a similar fold compared to that of PARM-pWT (Figure S2). While both Y459ψ and A461α yielded good kinetic data, only the latter variant had a sufficiently large effect on the binding energy to allow calculation of a Φ value. The A461α substitution yielded an intermediate Φ value of 0.50, which indicates that the probed backbone H-bond is partially formed in the transition state (Table S6). Kinetic data could not be obtained for I460ι as no signal change was observed in stopped flow experiments. Interestingly, D462δ resulted in a change from a linear correlation between *k*_obs_ and APP^WT^ concentration to a hyperbolic behavior, normally indicative of a conformational change during the binding reaction (Figure S10B). However, weak fluorescence signals in the binding as well as displacement experiments precluded mechanistic interpretations of the kinetic data for this variant.

Applying the double-mutant cycle analysis allowed us to further quantify the binding energy of two backbone–backbone H-bonds, namely, those probed by the A-to-E variants G756γ/A461α and N759η/Y459ψ, respectively (Figure 5C and Table S6). In both cases, negative ΔΔΔ*G*_eq_ values of ∼0.5 kcal mol^–1^ were obtained, showing that these bonds are energetically unfavorable in the bound state of the APP–Mint2 interaction. The negative ΔΔΔ*G*_eq_ values could be interpreted as the energy cost of breaking more favorable H-bonds to water in the unbound state, as the total change in the number of H-bonds in a binding reaction is usually zero.^42^

## Discussion

The family of Mint proteins, and Mint2 in particular, has attracted interest for their potential role in diseases in the brain, primarily Alzheimer’s disease,^43,44^ but recently also autism.^45^ In all cases, the disease relevance of Mint proteins relates to their function as scaffolding proteins and interaction with disease relevant proteins. In the case of Alzheimer’s disease, the role of Mint2 is established,^21^ and very recently, we investigated the Mint2–APP interaction in great detail. In particular, we combined genetic and pharmacological approaches to demonstrate a facilitative role of Mint2 in Aβ formation and developed a potent, metabolically stable peptide inhibitor of the Mint–APP interaction.^22^ However, the dynamics of the Mint2–APP coupled binding and folding interaction are not well understood, especially the importance of internal regulation by the α-helical ARM linker covering the PTB binding pocket. Xie et al. reported an open–closed motion of Mint2 that is dependent on APP binding and further hypothesized a regulatory effect on APP processing.^19^ Here, we show that the helical ARM linker blocks access to the binding pocket via a fast open–closed pre-equilibrium (*k*_obs_ ≫ 50 s^–1^). From the difference in affinity between PARM and PTB (without ARM), we can estimate that the ARM decreases the binding affinity of the APP peptide by approximately 20–30-fold. In other words, the open state is accessible to APP binding approximately 1/30 of the time for an average PARM molecule (based on the difference in *k*_on_) values. Thus, destabilization of the ARM linker interaction would increase affinity, and phosphorylation-mediated regulation of the ARM linker has been proposed to regulate APP binding.^23^ However, using semisynthesis we substituted Y543 with phosphotyrosine and showed that phosphorylation of Y543 did not modulate the affinity of the PARM–APP interaction. Thus, it is not clear whether the ARM serves a regulatory role or merely protects the binding pocket from nonspecific interactions.

Upon binding to PARM, APP folds into an extended conformation involving a short α-helix and a β-strand. The coupled folding and binding reaction of IDPs has attracted a great deal of attention due to its strong prevalence in protein–protein interactions. The biophysics of binding-induced folding have previously been characterized using a physical organic chemistry approach, i.e., site-directed mutagenesis combined with kinetics, for a growing number of interactions.^36−40,46−48^ General principles start to emerge, and it is clear that coupled binding and folding to some extent share features with folding of globular proteins. For example, as observed for APP/PARM, native contacts (the ones found in the bound complex) generally form late and cooperatively along the reaction coordinate. However, there are exceptions like cMyb/KIX,^37^ which appears to contain much native structure in the transition state. Another interaction, that between YAP and TEAD, shows features of a diffusion–collision mechanism^46^ in which certain structural elements form before others, reminiscent of the behavior of folding within the homeodomain family of globular proteins.^49^ Furthermore, the WASP GBD–Cdc42,^15^ cMyb–KIX,^13^ and CID–NCBD^12,50^ protein–protein interactions display templated folding, where malleability is observed in both transition and ground states and where the structured binding partner dictates the folding. Templated folding has been proposed as a general mechanism for binding-induced folding of IDPs,^14^ with a variable degree of plasticity in transition and ground states.

In this study, the LFERs and Φ values show that the mechanism of coupled binding and folding of APP to the PTB domain is robust to deletion or mutation of the ARM domain (Figures 4A–D and 5A and Figure S7) but that point mutations at positions F764 and F765 may change the binding interface and the transition state structure. Experimental Φ values show that the folding nucleus involves fractional native formation of the α-helical part of the bound APP peptide and that most of the β-strand forms downhill of the major transition state. The interaction differs from those of other IDPs in the unusually strong dependence on hydrophobic contacts driving the binding and also in a relatively high number of negative Φ values, which are consistent with non-native transition state constants (but can also be explained by ground state effects). The prevalence of hydrophobic contacts is probably facilitated by the ARM linker, which shields the binding pocket of the PTB domain in the apo state, thus protecting it from nonspecific hydrophobic interactions with other proteins. Non-native and native hydrophobic interactions may then promote formation of an initial encounter complex, which can rearrange as the reaction proceeds. This malleability of both the ground and transition state is consistent with templated folding. In conclusion, the LFERs and Φ values suggest that the coupled binding and folding reaction involves a transition state with a fractional contact formation very similar to that observed in protein folding studies (Brønsted β = 0.3) and in agreement with a nucleation–condensation mechanism, i.e., concomitant formation of secondary and tertiary structure as the transition state barrier is crossed.^34^
