# Supplementary material for: Molecular Details of a Coupled Binding and Folding Reaction between the Amyloid Precursor Protein and a Folded Domain
Source: ACS Chem Biol. 2021 Jun 23;16(7):1191–200. doi: 10.1021/acschembio.1c00176 (PMC8291497; doi:10.1021/acschembio.1c00176)
Supplement: Supplementary file 1 — cb1c00176_si_001.pdf [file cb1c00176_si_001.pdf]

Supporting information

Molecular details of a coupled binding and folding reaction between the amyloid precursor protein and a folded domain

Thomas M. T. Jensen,[†‡] Christian R. O. Bartling,[†] O. Andreas Karlsson,[‡] Emma Åberg,[‡] Linda M. Haugaard-Kedström,[†] Kristian Strømgaard[†]* and Per Jemth[‡]*

[†]Center for Biopharmaceuticals, Department of Drug Design and Pharmacology, University of Copenhagen, Jagtvej 162, 2100 Copenhagen, Denmark

[‡]Department of Medical Biochemistry and Microbiology, Uppsala University, BMC, Box 582, SE-75123 Uppsala, Sweden

Figure S1. The effect of the Y524W mutation in PARM and dansylation of APP. -- 3

Figure S2. Circular dichroism (CD) spectra of Mint2 PTB and PARM A-to-E variants. -- 5

Figure S3. FP saturation curves of APP binding to Mint2 PTB variants. -- 4

Figure S4. Stopped flow spectroscopy data for binding of APP variants. --- 6

Figure S5. Correlation between K_i and K_d values from FP and stopped flow (SF), respectively. ------------------------------ 8

Figure S6. Isothermal titration calorimetry measurements for APP binding to PARM and PTB. ------------------------------ 9

Figure S7. Correlation between k_{on} or k_{off} from the protein variants versus k_{on} or k_{off} from PARM, respectively.---------10

Figure S8. Characterization of semi synthetic PTB variants by SDS-PAGE. ---11

Figure S9. Hydrophobicity and free energy relationship of protein-protein interactions involving IDPs. -------------------12

Figure S10. Kinetic plots of A-to-E variants---14

Table S1. Characterization of synthesized peptides. --16

Table S2. Characterization of protein variants.---15

Table S3. Kinetic rate constants and calculated K_d from stopped flow measurements.--17

Table S4. K_i values measured by FP competition assays. ---18

Table S5. Calculated $\Delta\Delta G_{eq}$, $\Delta\Delta G_{TS}$ and Φ values.--19

Table S6. Kinetic characterization of APP and PARM A-to-E variants. --20

Supporting References---21

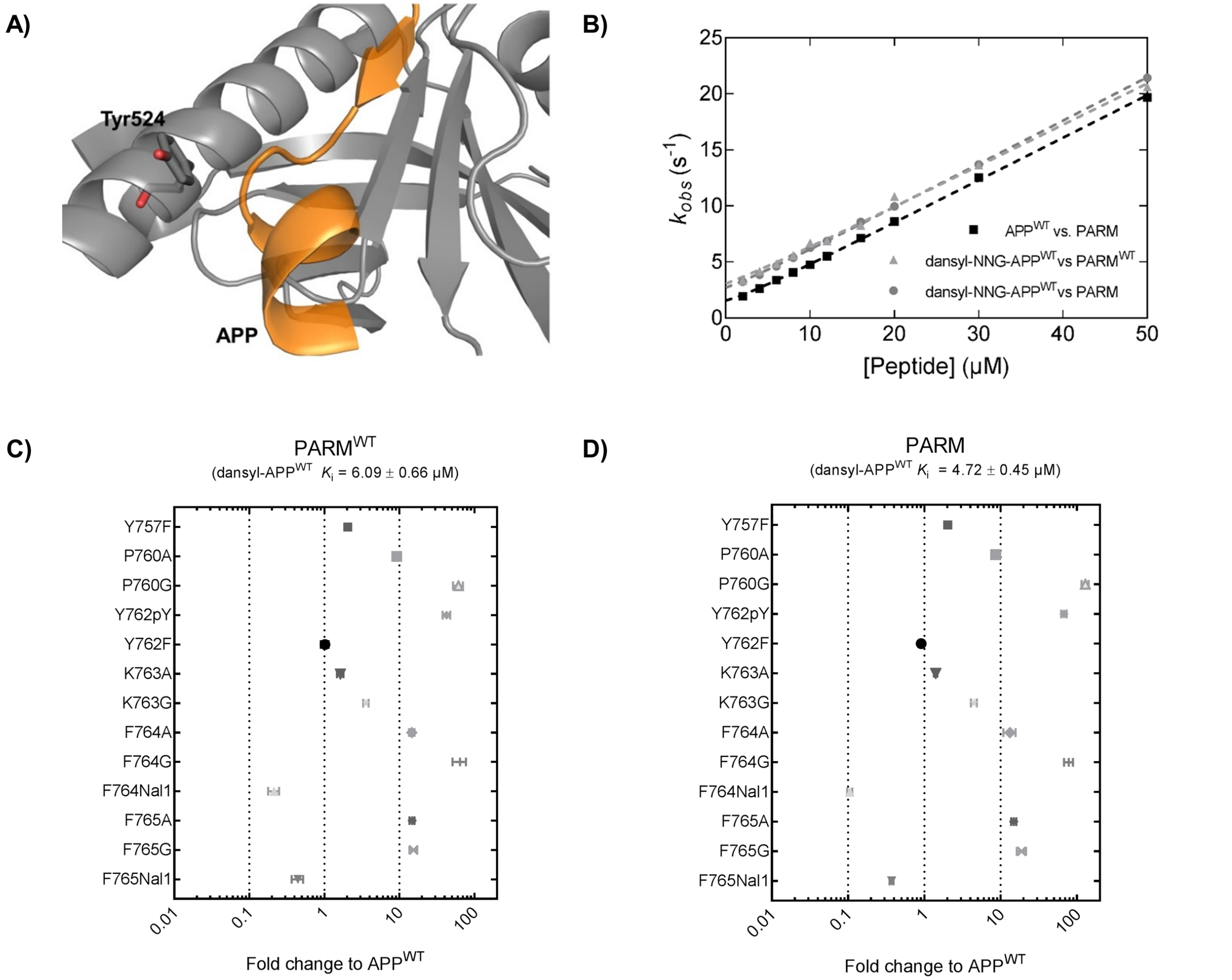

Figure S1. The effect of the Y524W mutation in PARM and dansylation of APP. (A) Cartoon representation of the interaction between APP (orange) and PARM (gray) highlighting the Y524 position (shown in sticks) used to introduce tryptophan to enable stopped flow measurements. (B) The observed rate constant (k_{obs}) was plotted against the concentration of peptide ligand. Both the introduced tryptophan (Y524W) in PARM (k_{on} = 0.39 ± 0.004 μM^{-1} s^{-1}, k_{off} = 2.3 ± 0.08 s^{-1}) and dansylation of APP^{WT} (k_{on} = 0.37 ± 0.009 μM^{-1} s^{-1}, k_{off} = 2.7 ± 0.18 s^{-1}) display similar kinetic behavior compared to the APP^{WT}/$PARM^{WT}$ interaction (k_{on} = 0.38 ± 0.003 μM^{-1} s^{-1}, k_{off} = 1.2 ± 0.05 s^{-1}). (C) Fold change in K_i values for dansyl-APP substitutions relative to dansyl-APP^{WT} for the binding to $PARM^{WT}$ measured by FP [K_i(variant)/K_i(WT) ± s.e.m]. (D) Fold change in K_i values for dansyl-APP substitutions relative to dansyl-APP^{WT} for the binding to PARM measured by FP [K_i(variant)/K_i(WT) ± s.e.m].

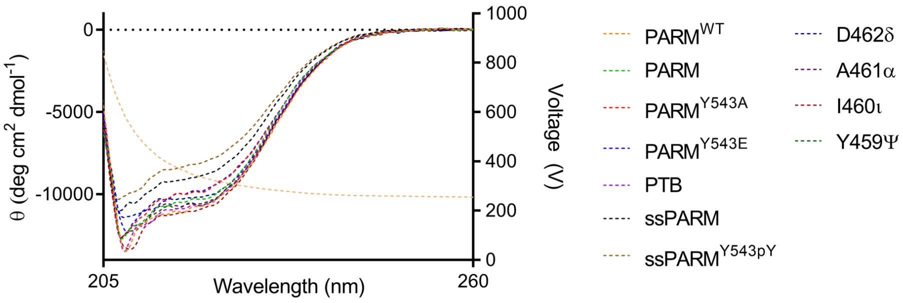

Figure S2. Circular dichroism (CD) spectra of Mint2 PTB and PARM A-to-E variants. CD spectra recorded using 15 µM protein in 500 mM NaCl, 25 mM HEPES, pH 7.4 at 25°C from 260-205 nm in mean residue ellipticity (deg cm^2 dmol^{-1}) (n=3). The applied voltage when testing PARM is shown on the right axis.

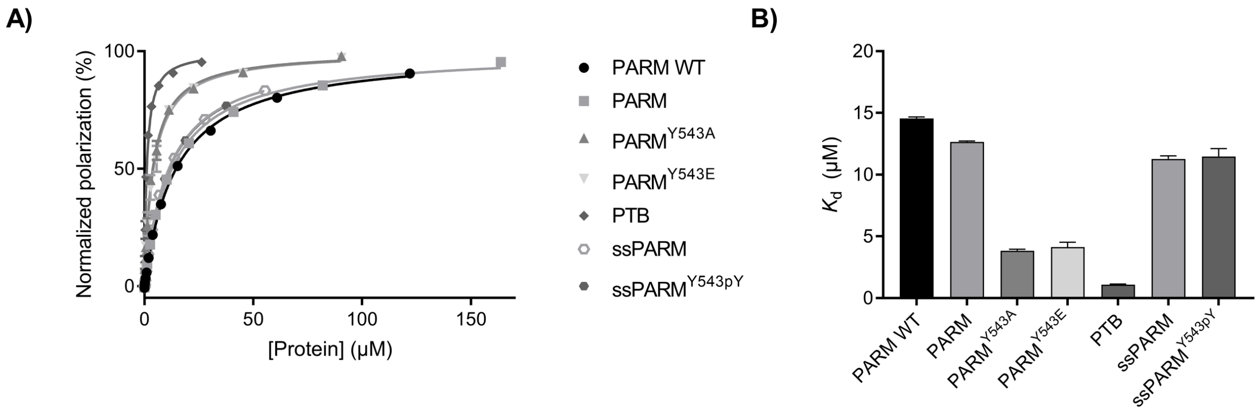

Figure S3. FP saturation curves of APP binding to Mint2 PTB variants. (A) FP saturation curves of the binding of TAMRA-NNG-NGYENPTYKFFE to PARM 364-570, PARM 364-570 (Y524W), PARM 364-570 (Y524W, Y543A), PARM 364-570 (Y524W, Y543E), PTB 364-538, ssPARM 364-560 (I533C, Y524W) and $ssPARM^{Y543pY}$ 364-560 (I533C, Y524W, Y543pY). (B) K_d values of APP binding to the Mint2 PTB variants (mean ± s.e.m., n=3).

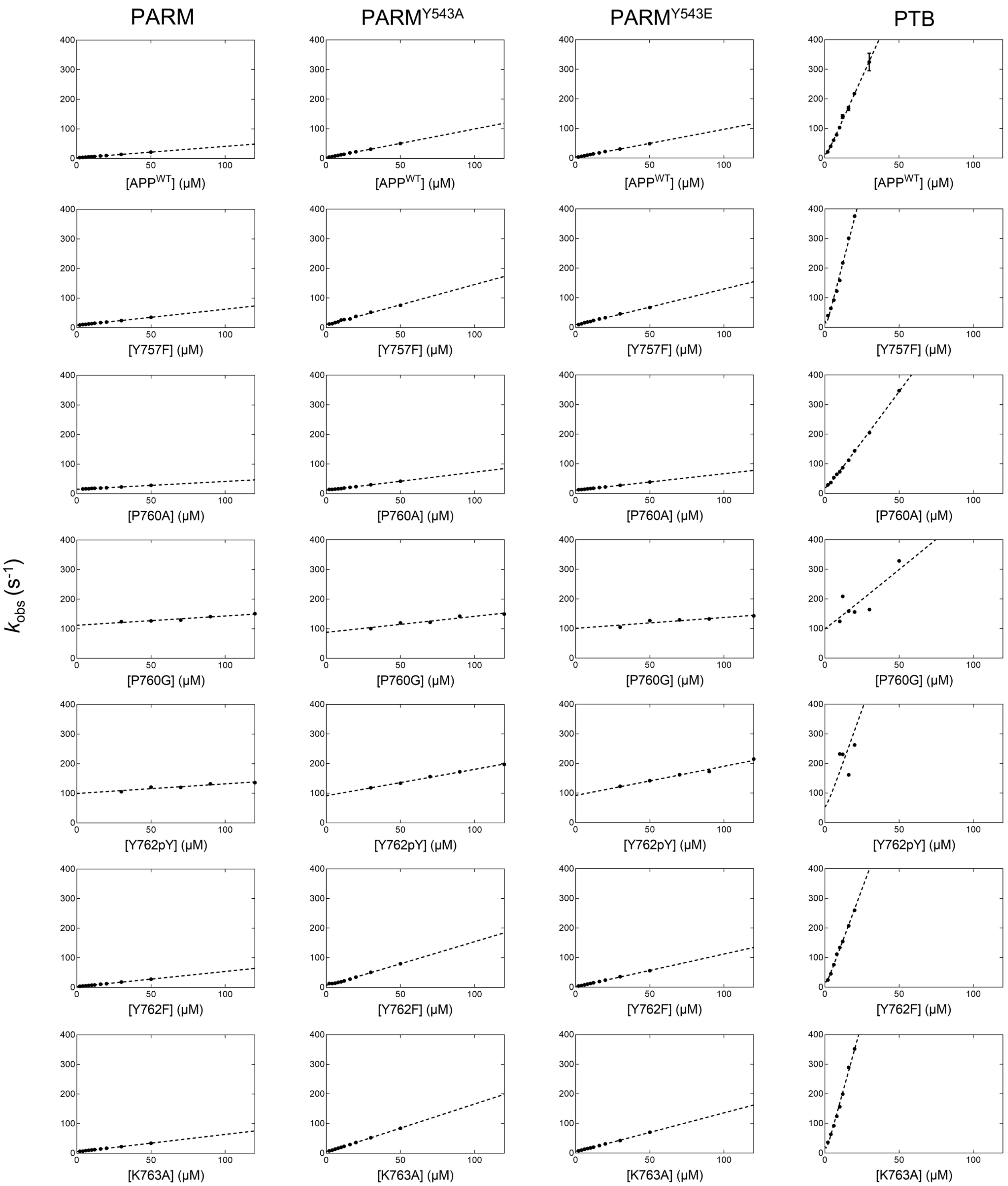
PARM
PARMY543A
PARMY543E
PTB
kobs (s-1)
[APPWT] (μM)
[Y757F] (μM)
[P760A] (μM)
[P760G] (μM)
[Y762pY] (μM)
[Y762F] (μM)
[K763A] (μM)

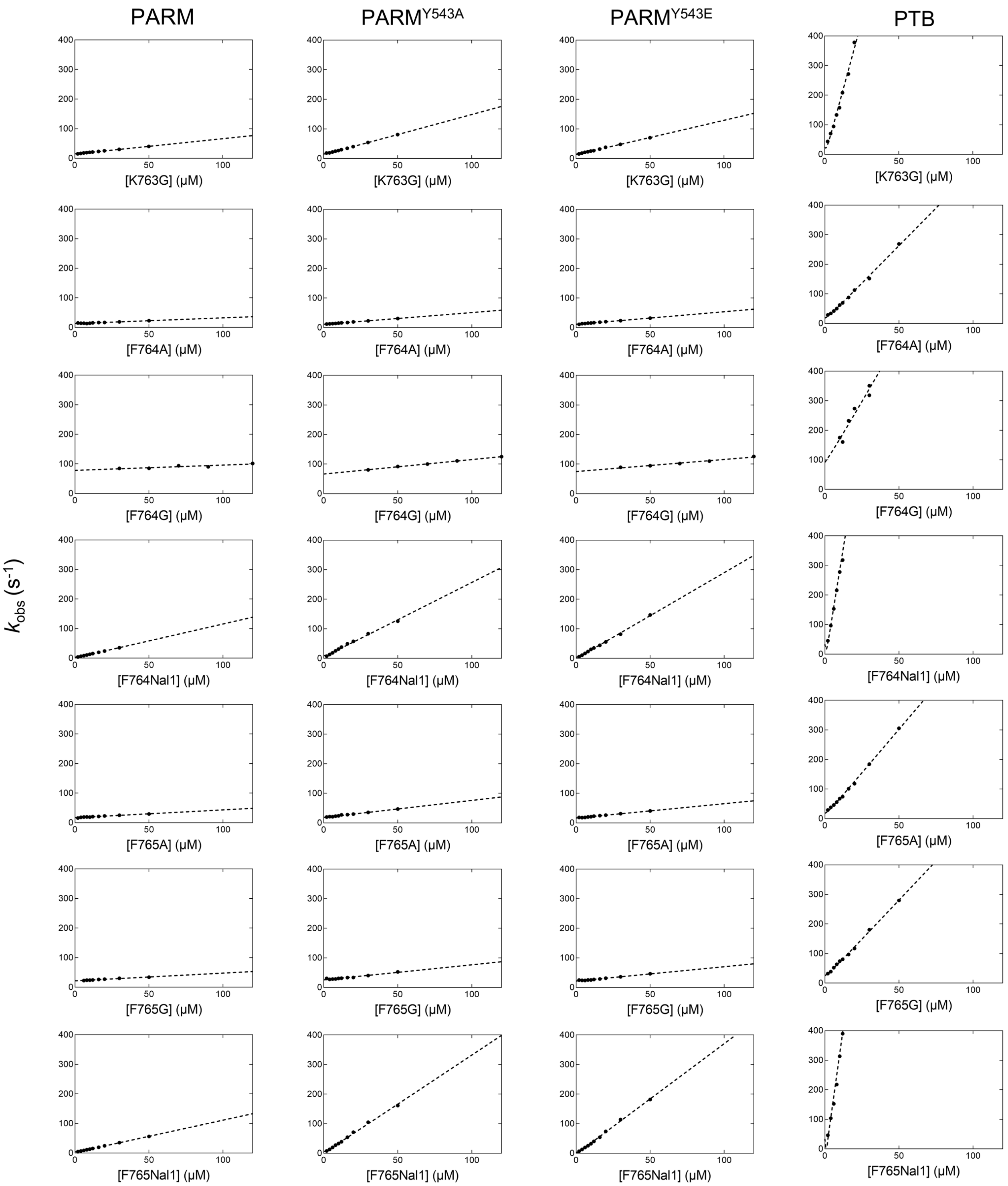

Figure S4. Stopped flow spectroscopy data for binding of APP variants. Data were recorded using 1 μM protein in 500 mM NaCl, 25 mM HEPES, pH 7.4, 25°C. The observed rate constants (k_{obs}) were plotted against the peptide concentration and fitted to the general equation for a reversible bimolecular interaction (see methods).

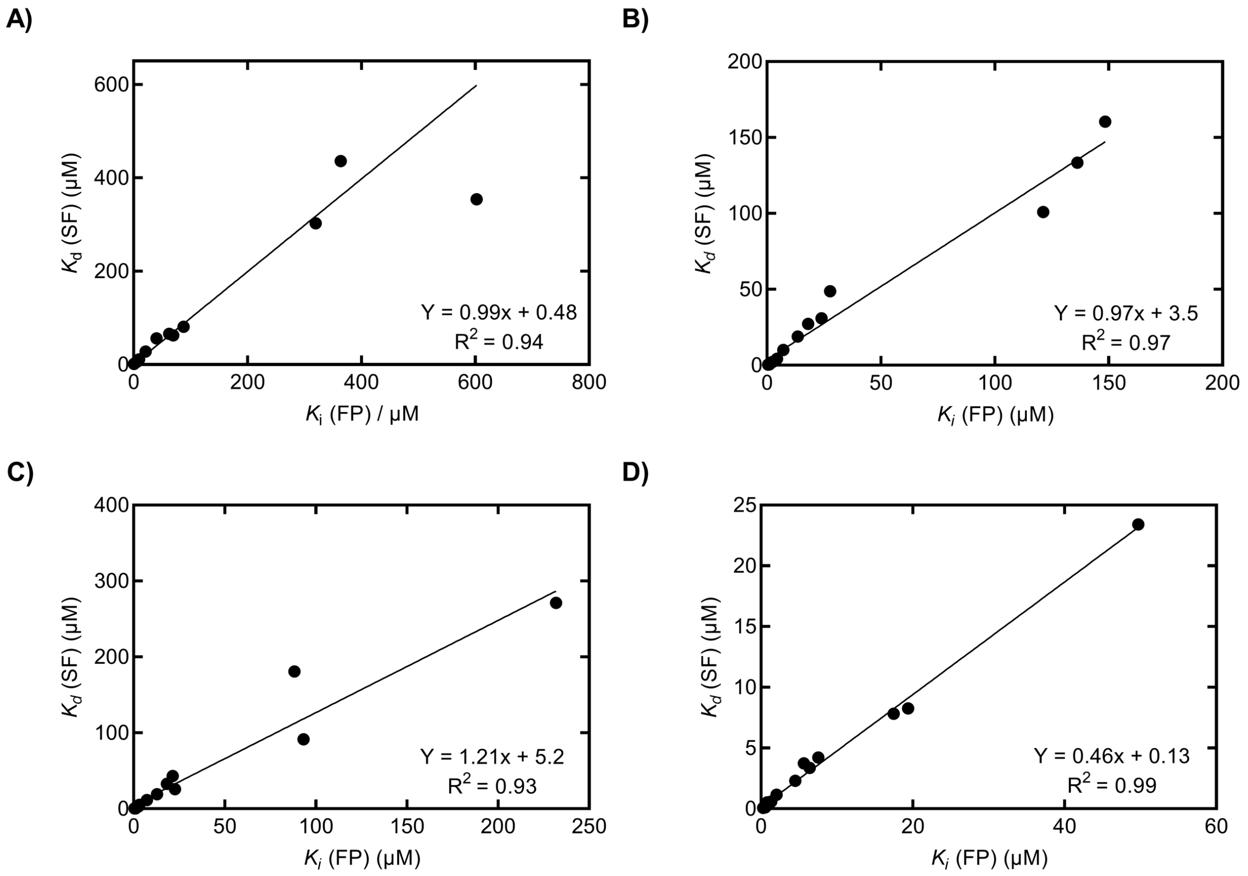

Figure S5. Correlation between K_i and K_d values from FP and stopped flow (SF), respectively. The calculated K_d values from stopped flow measurements are plotted against the respective K_i value measured by FP and fitted to a straight line. (A) Dansyl-APP variants against PARM. (B) Dansyl-APP variants against $PARM^{Y543A}$. (C) Dansyl-APP variants against $PARM^{Y543E}$. (D) Dansyl-APP variants against PTB. From the slope it can be seen that there is a good correlation between FP and SF, but the affinity determined by FP compared to SF is two times higher.

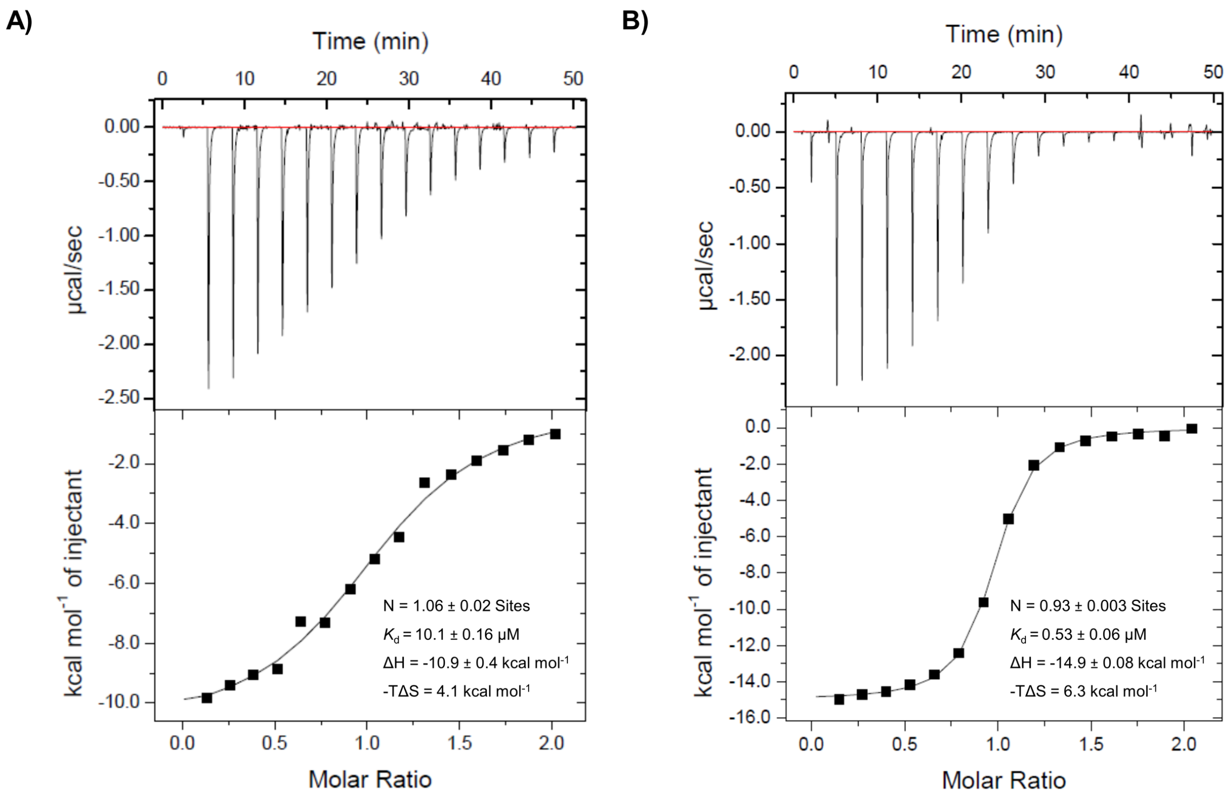

Figure S6. Isothermal titration calorimetry measurements for APP binding to PARM and PTB. For comparison with FP and stopped flow data, the same buffer conditions were used (500 mM NaCl, 25 mM HEPES, pH 7.4, 25°C). (A) Titration of dansyl-APP^{WT} against PARM. (B) Titration of dansyl-APP^{WT} against PTB.

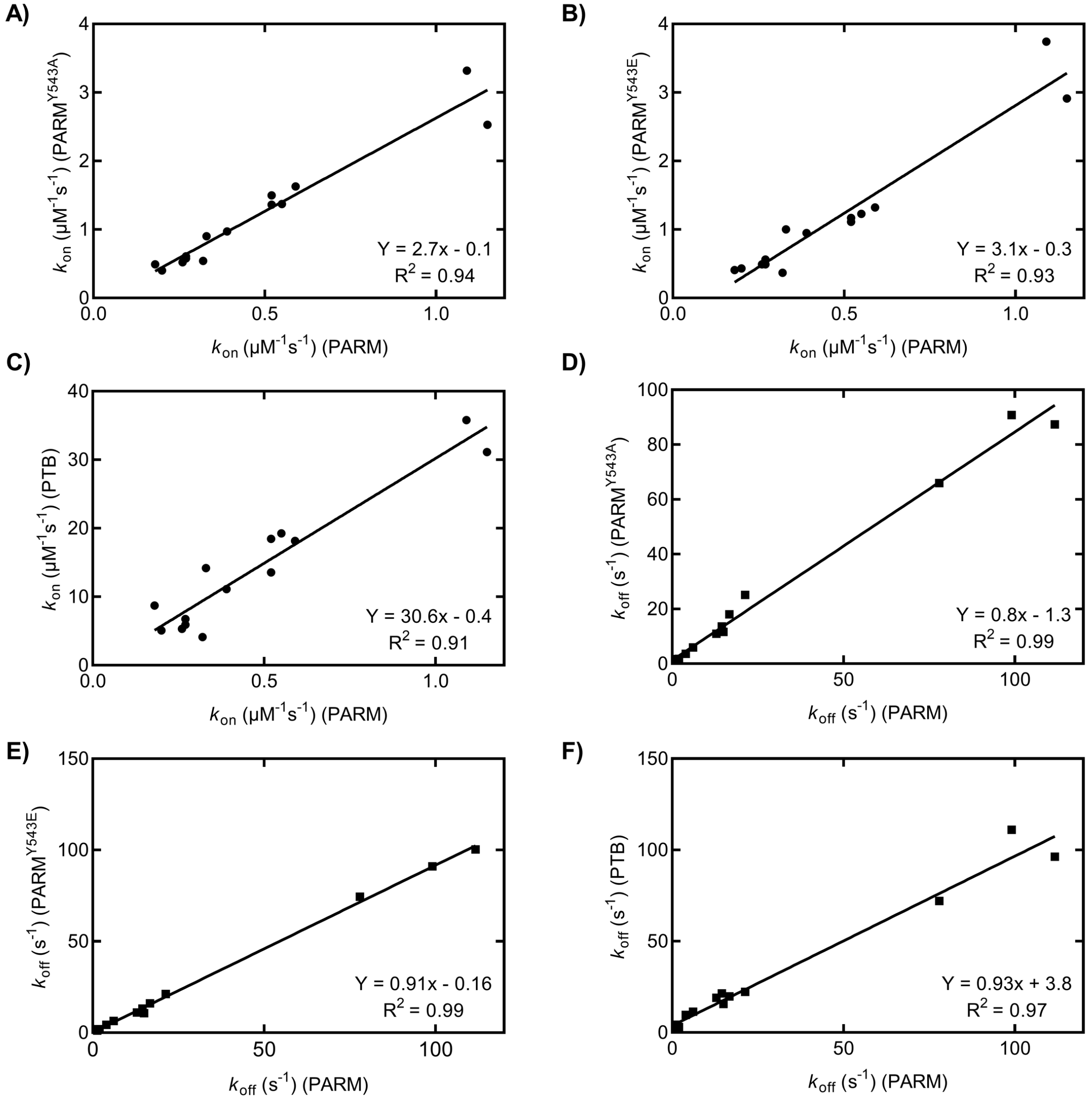

Figure S7. Correlation between k_{on} or k_{off} from the protein variants versus k_{on} or k_{off} from PARM, respectively. The k_{on} values of dansyl-APP variant towards A) PARM^{Y543A}, B) PARM^{Y543E}and C) PTB are plotted against the respective k_{on} values of dansyl-APP variants for the binding to PARM and fitted to a straight line. Likewise, the k_{off} values of dansyl-APP variants towards D) PARM^{Y543A}, E) PARM^{Y543E}and F) PTB are plotted against the respective k_{off} values of dansyl-APP variants for the binding to PARM and fitted to a straight line.

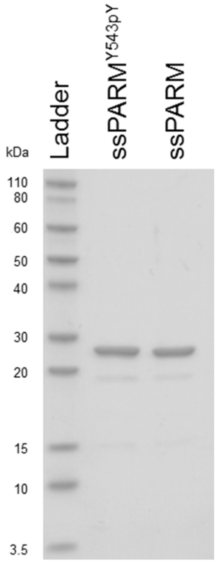

Figure S8. Characterization of semi synthetic PTB variants by SDS-PAGE. 16% RunBlue SDS-PAGE of ssPARM and ssPARM^{Y543pY}.

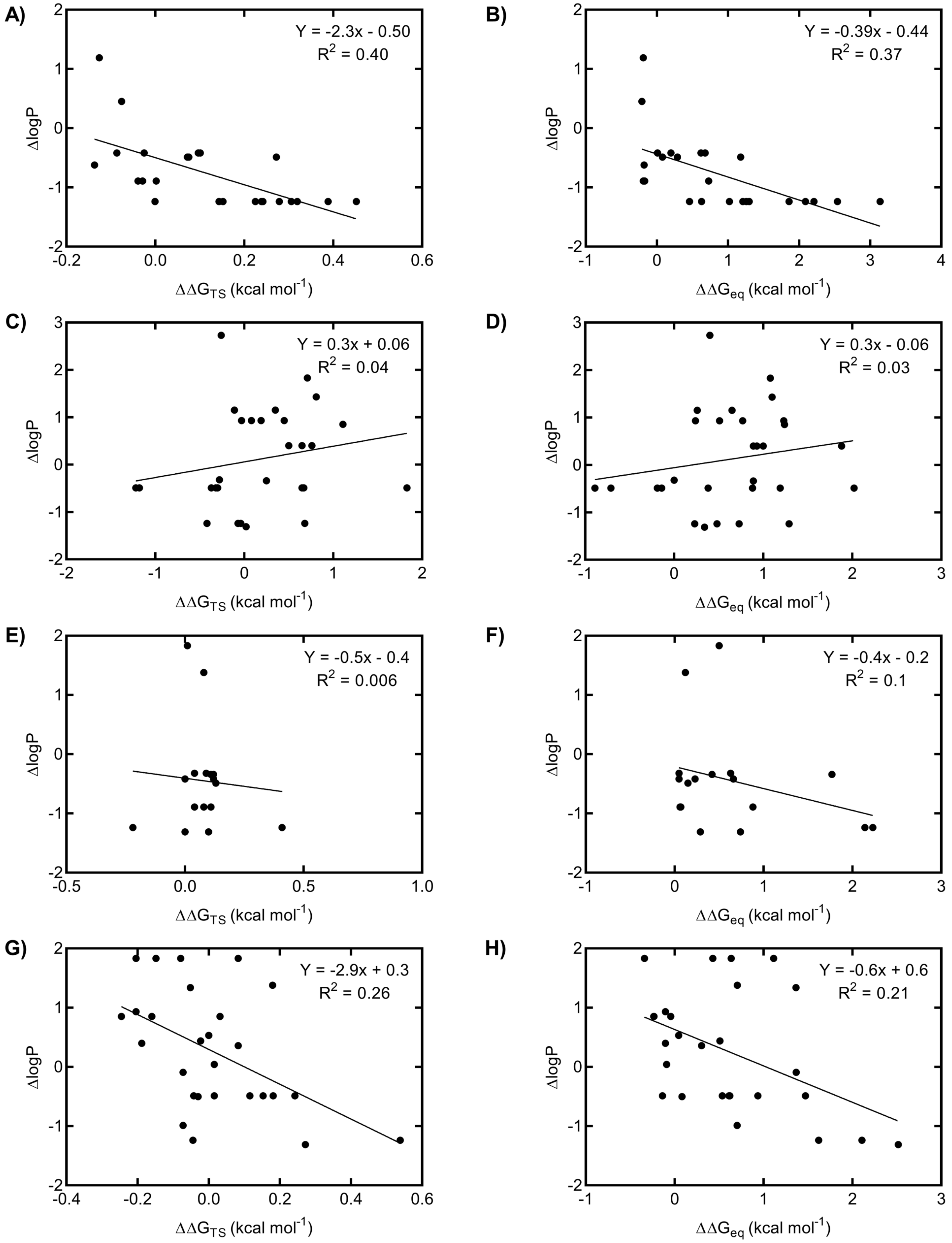
A)
Y = -2.3x - 0.50
R^2 = 0.40
ΔlogP
ΔΔG_TS (kcal mol^-1)
B)
Y = -0.39x - 0.44
R^2 = 0.37
ΔlogP
ΔΔG_eq (kcal mol^-1)
C)
Y = 0.3x + 0.06
R^2 = 0.04
ΔlogP
ΔΔG_TS (kcal mol^-1)
D)
Y = 0.3x - 0.06
R^2 = 0.03
ΔlogP
ΔΔG_eq (kcal mol^-1)
E)
Y = -0.5x - 0.4
R^2 = 0.006
ΔlogP
ΔΔG_TS (kcal mol^-1)
F)
Y = -0.4x - 0.2
R^2 = 0.1
ΔlogP
ΔΔG_eq (kcal mol^-1)
G)
Y = -2.9x + 0.3
R^2 = 0.26
ΔlogP
ΔΔG_TS (kcal mol^-1)
H)
Y = -0.6x + 0.6
R^2 = 0.21
ΔlogP
ΔΔG_eq (kcal mol^-1)

Figure S9. Hydrophobicity and free energy relationship of protein-protein interactions involving IDPs. The change in hydrophobicity from the ligand (ΔLogP) was plotted versus the $\Delta\Delta G_{eq}$ or $\Delta\Delta G_{TS}$, respectively for the interaction of reported protein-protein interactions. ΔLogP (n-Octanol/Water) was calculated using ChemBio-Draw based on Crippens fragmentation[1]. (A,B) ACTR/NCBD.[2] (C,D) cMyb/KIX.[3] (E,F) STAT2/TAZ1.[4] (G,H) pKID/KIX.[5]

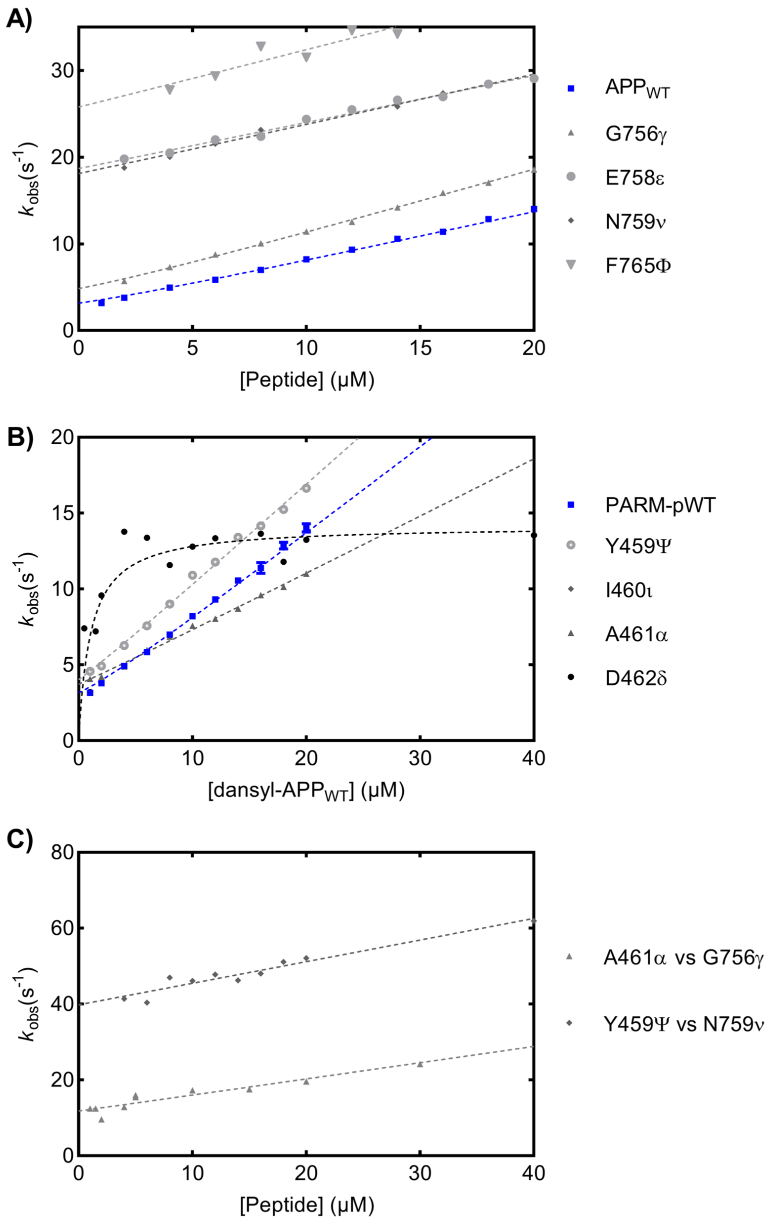

Figure S10. Kinetic plots of A-to-E variants. k_{obs} was plotted as a function of peptide concentration. (A) APP A-to-E variants. (B) PARM-pWT A-to-E variants. The k_{obs} values of D462δ shows a hyperbolic behavior, which could indicate a change in binding kinetics. However, data at low peptide concentration could not be obtained; hence the kinetic constants could not be calculated. (C) Kinetic plots of double mutant cycle of A-to-E substitutions in APP and PARM-pWT.

Table S1. Characterization of synthesized peptides.

Peptide	Sequence	Purity (%) (214 nm)	Calculated mass [M+H]	Observed mass [M+H]
TAMRA-APP^{WT}	TAMRA-NNG-NGYENPTYKFFE	> 95	2206.9	2207.8
dansyl-APP^{WT}	dansyl-NNG-NGYENPTYKFFE	> 95	2027.2	2026.2
dansyl-APP^{Y757F}	dansyl-NNG-NGFENPTYKFFE	> 95	2011.2	2010.2
dansyl-APP^{P760A}	dansyl-NNG-NGYENATYKFFE	> 95	2002.1	2001.2
dansyl-APP^{P760G}	dansyl-NNG-NGYENGTYKFFE	> 95	1987.1	1986.2
dansyl-APP^{Y762pY}	dansyl-NNG-NGYENPTpYKFFE	> 95	2107.1	2106.2
dansyl-APP^{Y762F}	dansyl-NNG-NGYENPTFKFFE	> 95	2011.2	2010.2
dansyl-APP^{K763A}	dansyl-NNG-NGYENPTYAFFE	> 95	1970.1	1969.2
dansyl-APP^{K763G}	dansyl-NNG-NGYENPTYGFFE	> 95	1956.0	1955.2
dansyl-APP^{F764A}	dansyl-NNG-NGYENPTYKAFE	> 95	1951.1	1950.2
dansyl-APP^{F764G}	dansyl-NNG-NGYENPTYKGFE	> 95	1937.0	1936.2
dansyl-APP^{F764Nal1}	dansyl-NNG-NGYENPTYKNal1FE	> 95	2077.2	2076.4
dansyl-APP^{F765A}	dansyl-NNG-NGYENPTYKFAE	> 95	1951.1	1950.2
dansyl-APP^{F765G}	dansyl-NNG-NGYENPTYKFGE	> 95	1937.0	1936.2
dansyl-APP^{F765Nal1}	dansyl-NNG-NGYENPTYKFNal1E	> 95	2077.2	2076.4
APP^{WT}	NGYENPTYKFFE	> 95	1508.7	1507.7
ARM^{WT}	CNPEDLSQKEYSDIINTQEMYNDDLIHF	> 90	3375.6	3374.7
ARM^{Y543pY}	CNPEDLSQKE(pY)SDIINTQEMYNDDLIHF	> 85	3455.6	3455.1
Dansyl-APP$^{G756\gamma}$	Dansyl-NNG-NγYENPTYKFFE	> 90	2028.2	2027.0
Dansyl-APP$^{Y757\psi}$	Dansyl-NNG-NGψENPTYKFFE	> 90	2028.2	2027.0
Dansyl-APP$^{E758\varepsilon}$	Dansyl-NNG-NGYεNPTYKFFE	> 90	2028.2	2028.0
Dansyl-APP$^{N759\nu}$	Dansyl)-NNG-NGYEνPTYKFFE	> 90	2028.2	2028.0
Dansyl-APP$^{F764\varphi}$	Dansyl-NNG-NGYENPTYKϕFE	> 90	2028.2	2028.2

Table S2. Characterization of protein variants.

Protein[1]	hMint2 Sequence	Purity (%) (214 nm)	Calculated mass [M+H]	Observed mass [M+H]
PARM^{WT} [2]	364-570	> 95	25,237	25,235
PARM[2]	364-570	> 95	25,261	25,259
PARM^{Y543A}(2)	364-570	> 95	25,168	25,168
PARM^{Y543E}(2)	364-570	> 95	25,226	25,225
PTB[2]	364-538	> 95	21,441	21,440
PTB-MesNa	364-532	> 90	19,180	19,179
ssPARM(3)	364-560	> 90	22,414	22,411
ssPARM^{Y543pY}(3)	364-560	> 90	22,494	22,491
PARM-pWT(4)	364-570	> 90	23,433	23,431
Y459ψ(4)	364-570	> 85	23,433	23,431
I460ι(4)	364-570	> 85	23,433	23,433
A461α(4)	364-570	75	23,433	23,431
D462δ(4)	364-570	> 90	23,433	23,431

[1]All proteins (except PARM^{WT}) include the Y524W mutation to enable fluorescence detection in stopped flow. [2]Contains an N-terminal His-tag. [3]Including I533C to facilitate semisynthesis.[4]Including R455K, C483A, C501A, C566A to facilitate A-to-E substitutions by semisynthesis.

Table S3. Kinetic rate constants and calculated K_d from stopped flow experiments.

Peptide	PARM k_{on} ($\mu M^{-1}s^{-1}$)	PARM k_{off} (s^{-1})	PARM K_d (μM)	PARM^{Y543A} k_{on} ($\mu M^{-1}s^{-1}$)	PARM^{Y543A} k_{off} (s^{-1})	PARM^{Y543A} K_d (μM)
APP^{WT}	0.39 ± 0.004	1.9 ± 0.1	5.0 ± 0.3	0.97 ± 0.01	1.8 ± 0.01	1.8 ± 0.02
Y757F	0.55 ± 0.01	6.1 ± 0.3	11 ± 0.6	1.4 ± 0.04	5.9 ± 0.12	4.3 ± 0.2
P760A	0.27 ± 0.01	15 ± 0.1	56 ± 2	0.61 ± 0.01	12 ± 0.23	19 ± 0.7
P760G	0.32 ± 0.05	112 ± 4	354 ± 63	0.54 ± 0.08	87 ± 6.51	160 ± 36
Y762pY	0.33 ± 0.06	99 ± 5	302 ± 74	0.90 ± 0.03	91 ± 2	101 ± 5
Y762F	0.52 ± 0.005	2.1 ± 0.04	4.0 ± 0.08	1.5 ± 0.04	1.8 ± 0.04	1.2 ± 0.04
K763A	0.59 ± 0.01	4.0 ± 0.2	6.7 ± 0.3	1.6 ± 0.01	3.6 ± 0.11	2.2 ± 0.07
K763G	0.52 ± 0.01	15 ± 0.2	28 ± 0.8	1.4 ± 0.01	14 ± 0.26	10 ± 0.3
F764A	0.20 ± 0.02	13 ± 0.4	66 ± 8	0.40 ± 0.005	11 ± 0.1	27 ± 0.6
F764G	0.18 ± 0.05	78 ± 4	436 ± 139	0.49 ± 0.01	66 ± 0.76	133 ± 4
F764Nal1	1.2 ± 0.01	1.3 ± 0.03	1.1 ± 0.03	2.5 ± 0.08	1.3 ± 0.02	0.49 ± 0.02
F765A	0.27 ± 0.02	18 ± 0.4	62 ± 5	0.58 ± 0.02	18 ± 0.38	31 ± 2
F765G	0.26 ± 0.02	21 ± 0.4	81 ± 6	0.52 ± 0.04	25 ± 0.85	47 ± 5
F765Nal1	1.1 ± 0.01	1.6 ± 0.02	1.5 ± 0.02	3.3 ± 0.06	1.7 ± 0.04	0.50 ± 0.01

Peptide	PARM^{Y543E} k_{on} ($\mu M^{-1}s^{-1}$)	PARM^{Y543E} k_{off} (s^{-1})	PARM^{Y543E} K_d (μM)	PTB k_{on} ($\mu M^{-1}s^{-1}$)	PTB k_{off} (s^{-1})	PTB K_d (μM)
APP^{WT}	0.95 ± 0.01	1.8 ± 0.01	1.9 ± 0.03	11 ± 0.2	3.1 ± 0.02	0.28 ± 0.01
Y757F	1.2 ± 0.03	6.4 ± 0.14	5.2 ± 0.2	19 ± 0.6	12 ± 0.3	0.60 ± 0.02
P760A	0.56 ± 0.003	11 ± 0.05	19 ± 0.2	6.8 ± 0.07	16 ± 0.3	2.3 ± 0.05
P760G	0.37 ± 0.09	100 ± 7	271 ± 85	4.1 ± 1	96 ± 27	23 ± 10
Y762pY	1.0 ± 0.08	91 ± 6	91 ± 13	14 ± 5	111 ± 8	7.8 ± 3
Y762F	1.1 ± 0.01	1.8 ± 0.01	1.6 ± 0.02	14 ± 0.2	2.9 ± 0.1	0.22 ± 0.01
K763A	1.3 ± 0.01	4.3 ± 0.05	3.2 ± 0.05	18 ± 0.4	9.7 ± 0.6	0.53 ± 0.04
K763G	1.2 ± 0.02	13 ± 0.4	11 ± 0.4	18 ± 0.7	22 ± 0.5	1.2 ± 0.05
F764A	0.43 ± 0.01	11 ± 0.3	26 ± 1	5.1 ± 0.1	19 ± 0.8	3.7 ± 0.2
F764G	0.41 ± 0.03	74 ± 2	181 ± 18	8.7 ± 1	72 ± 2	8.3 ± 1
F764Nal1	2.9 ± 0.04	1.2 ± 0.01	0.43 ± 0.007	31 ± 1	2.3 ± 0.09	0.07 ± 0.004
F765A	0.49 ± 0.01	16 ± 0.3	33 ± 2	5.9 ± 0.09	20 ± 0.8	3.4 ± 0.2
F765G	0.49 ± 0.02	21 ± 0.5	43 ± 3	5.3 ± 0.08	22 ± 0.5	4.2 ± 0.1
F765Nal1	3.7 ± 0.05	2.0 ± 0.04	0.53 ± 0.01	36 ± 1	4.3 ± 0.02	0.12 ± 0.004

See Methods section in main text for errors.

Table S4. ***K*$_i$ values measured by FP competition assays.**

Peptide	PARM^{WT}	PARM	PARM^{Y543A}	PARM^{Y543E}	PTB
APP^{WT}	6.1 ± 0.7	4.7 ± 0.3	1.7 ± 0.2	1.8 ± 0.1	0.81 ± 0.06
Y757F	13 ± 1	9.5 ± 0.2	4.4 ± 0.7	3.1 ± 0.1	1.4 ± 0.16
P760A	55 ± 0.7	40 ± 0.8	14 ± 1	13 ± 0.1	4.5 ± 0.19
P760G	362 ± 38	602 ± 35	149 ± 12	232 ± 31	49 ± 4
Y762pY	253 ± 4	320 ± 30	121 ± 3	93 ± 0.9	18 ± 2
Y762F	6.1 ± 0.4	4.3 ± 0.1	1.7 ± 0.3	1.6 ± 0.08	0.64 ± 0.04
K763A	9.7 ± 0.04	6.6 ±0.2	2.5 ± 0.06	2.7 ± 0.1	0.80 ± 0.009
K763G	21 ± 1	21 ± 1	7.1 ± 0.3	7.2 ± 0.2	2.1 ± 0.09
F764A	88 ± 4	63 ± 11	18 ± 3	23 ± 0.6	5.6 ± 0.7
F764G	375 ± 42	364 ± 30	136 ± 12	88 ± 5	19 ± 4
F764Nal1	1.3 ± 0.2	0.5 ± 0.05	0.5 ± 0.1	0.47 ± 0.03	0.29 ± 0.02
F765A	89 ± 5	68 ± 3	24 ± 0.8	18 ± 0.6	6.4 ± 0.4
F765G	92 ± 6	88 ± 7	28 ± 0.5	21 ± 0.3	7.5 ± 0.5
F765Nal1	2.6 ± 0.2	1.8 ± 0.02	1.0 ± 0.3	1.1 ± 0.09	0.56 ± 0.02
APP^{WT} (12-mer)	3.2 ± 0.6	3.5 ± 0.9	1.1 ± 0.02	1.1 ± 0.01	0.74 ± 0.03

All K_d values are reported in µM and are a mean ± s.e.m, n =3.

Table S5. Calculated ΔΔG_{eq}, ΔΔG_{TS} and Φ values.

Peptide	PARM ΔΔG_{eq} (kcal mol^{-1})	PARM ΔΔG_{TS} (kcal mol^{-1})	PARM Φ	PARM^{Y543A} ΔΔG_{eq} (kcal mol^{-1})	PARM^{Y543A} ΔΔG_{TS} (kcal mol^{-1})	PARM^{Y543A} Φ
Y757F	0.48 ± 0.04	-0.20 ± 0.003	-0.43 ± 0.03	0.52 ± 0.02	-0.20 ± 0.007	-0.39 ± 0.02
P760A	1.4 ± 0.09	0.22 ± 0.006	0.16 ± 0.01	1.4 ± 0.05	0.27 ± 0.006	0.20 ± 0.01
P760G	2.5 ± 0.5	0.12 ± 0.02	0.05 ± 0.01	2.7 ± 0.6	0.34 ± 0.05	0.13 ± 0.04
Y762pY	2.4 ± 0.6	0.10 ± 0.02	0.04 ± 0.01	2.4 ± 0.01	0.05 ± 0.002	0.02 ± 0.001
Y762F	-0.14 ± 0.01	-0.17 ± 0.002	-	-0.23 ± 0.01	-0.25 ± 0.007	-
K763A	0.18 ± 0.01	-0.25 ± 0.004	-	0.13 ± 0.004	-0.31 ± 0.004	-
K763G	1.0 ± 0.06	-0.17 ± 0.003	-0.17 ± 0.01	1.02 ± 0.03	-0.20 ± 0.003	-0.20 ± 0.01
F764A	1.5 ± 0.2	0.41 ± 0.04	0.27 ± 0.04	1.6 ± 0.04	0.52 ± 0.009	0.33 ± 0.01
F764G	2.7 ± 0.9	0.46 ± 0.1	0.17 ± 0.07	2.6 ± 0.09	0.40 ± 0.009	0.16 ± 0.01
F764Nal1	-0.88 ± 0.05	-0.64 ± 0.008	0.73 ± 0.04	-0.77 ± 0.03	-0.56 ± 0.02	0.74 ± 0.04
F765A	1.5 ± 0.1	0.22 ± 0.01	0.15 ± 0.02	1.7 ± 0.09	0.30 ± 0.01	0.18 ± 0.01
F765G	1.7 ± 0.2	0.23 ± 0.01	0.14 ± 0.02	2.0 ± 0.21	0.38 ± 0.03	0.19 ± 0.03
F765Nal1	-0.73 ± 0.04	-0.61 ± 0.008	0.84 ± 0.05	-0.75 ± 0.02	-0.73 ± 0.02	0.96 ± 0.04
A760G	1.1 ± 0.2	-0.1 ± 0.02	-0.09 ± 0.02	1.3 ± 0.3	0.072 ± 0.01	0.06 ± 0.02
A763G	0.84 ± 0.04	0.07 ± 0.02	0.09 ± 0.005	0.89 ± 0.04	0.11 ± 0.001	0.12 ± 0.005
A764G	1.1 ± 0.4	0.05 ± 0.02	0.05 ± 0.02	0.94 ± 0.03	-0.12 ± 0.003	-0.13 ± 0.01
A765G	0.15 ± 0.02	0.008 ± 0.001	-	0.27 ± 0.03	0.07 ± 0.006	-

Peptide	PARM^{Y543E} ΔΔG_{eq} (kcal mol^{-1})	PARM^{Y543E} ΔΔG_{TS} (kcal mol^{-1})	PARM^{Y543E} Φ	PTB ΔΔG_{eq} (kcal mol^{-1})	PTB ΔΔG_{TS} (kcal mol^{-1})	PTB Φ
Y757F	0.59 ± 0.02	-0.15 ± 0.004	-0.25 ± 0.01	0.45 ± 0.02	-0.33 ± 0.01	-0.72 ± 0.04
P760A	1.4 ± 0.03	0.31 ± 0.005	0.23 ± 0.01	1.3 ± 0.04	0.29 ± 0.006	0.23 ± 0.01
P760G	2.9 ± 0.9	0.56 ± 0.1	0.19 ± 0.08	2.6 ± 1.1	0.59 ± 0.2	0.22 ± 0.1
Y762pY	2.3 ± 0.3	-0.03 ± 0.002	-0.01 ± 0.002	2.0 ± 0.7	-0.14 ± 0.05	-0.07 ± 0.03
Y762F	-0.12 ± 0.003	-0.09 ± 0.002	-	-0.15 ± 0.01	-0.12 ± 0.003	-
K763A	0.31 ± 0.01	-0.19 ± 0.003	-	0.39 ± 0.03	-0.29 ± 0.009	-0.75 ± 0.06
K763G	1.1 ± 0.04	-0.12 ± 0.003	-0.11 ± 0.01	0.85 ± 0.04	-0.30 ± 0.01	-0.35 ± 0.02
F764A	1.5 ± 0.08	0.47 ± 0.02	0.31 ± 0.02	1.5 ± 0.07	0.46 ± 0.01	0.30 ± 0.02
F764G	2.7 ± 0.3	0.50 ± 0.04	0.18 ± 0.02	2.0 ± 0.3	0.14 ± 0.018	0.07 ± 0.01
F764Nal1	-0.89 ± 0.02	-0.66 ± 0.01	0.74 ± 0.02	-0.79 ± 0.04	-0.61 ± 0.024	0.77 ± 0.05
F765A	1.7 ± 0.08	0.39 ± 0.02	0.23 ± 0.01	1.5 ± 0.07	0.37 ± 0.009	0.25 ± 0.01
F765G	1.6 ± 0.1	0.39 ± 0.02	0.25 ± 0.02	1.6 ± 0.06	0.44 ± 0.01	0.27 ± 0.01
F765Nal1	-0.8 ± 0.02	-0.81 ± 0.02	1.1 ± 0.04	-0.50 ± 0.02	-0.69 ± 0.03	1.4 ± 0.08
A760G	1.6 ± 0.5	0.25 ± 0.06	0.16 ± 0.06	1.5 ± 0.7	0.3 ± 0.1	0.2 ± 0.11
A763G	0.74 ± 0.03	0.074 ± 0.001	0.10 ± 0.004	0.46 ± 0.04	-0.009 ± 0.0004	-0.02 ± 0.002
A764G	1.2 ± 0.13	0.025 ± 0.002	0.02 ± 0.003	0.47 ± 0.06	-0.32 ± 0.04	-0.7 ± 0.13
A765G	0.16 ± 0.01	0.0006 ± 0.00004	-	0.13 ± 0.01	0.06 ± 0.001	-

See Methods section in main text for errors.

Table S6. Parameters derived from the kinetic characterization of APP and PARM A-to-E variants.

			Dansyl-APP^{WT}			
**PARM**	k_{on} (μM^{-1}s^{-1})	k_{off} (s^{-1})	K_d (μM)	ΔΔG_{eq} (kcal mol^{-1})	ΔΔG_{TS} (kcal mol^{-1})	Φ
pWT	0.57 ± 0.003	2.5 ± 0.01	4.3 ± 0.02	-	-	-
Y459ψ	0.67 ± 0.02	3.4 ± 0.1	5.0 ± 0.2	0.09 ± 0.004	-0.09 ± 0.002	-
I460ι	-	-	-	-	-	-
A461α	0.38 ± 0.01	3.8 ± 0.2	9.8 ± 0.6	0.49 ± 0.03	0.25 ± 0.01	0.50 ± 0.03
D462δ	-	-	-	-	-	-
			**PARM-pWT**			
**APP**	k_{on} (μM^{-1}s^{-1})	k_{off} (s^{-1})	K_d (μM)	ΔΔG_{eq} (kcal mol^{-1})	ΔΔG_{TS} (kcal mol^{-1})	Φ
G756γ	0.75 ± 0.01	3.4 ± 0.08	4.6 ± 0.12	0.04 ± 0.001	-0.16 ± 0.002	-
Y757ψ	-	-	-	-	-	-
E758ε	0.56 ± 0.02	18.1 ± 0.3	33 ± 1.7	1.2 ± 0.06	0.02 ± 0.001	0.01 ± 0.001
N759η	0.6 ± 0.02	14.6 ± 0.3	24 ± 1.1	1.03 ± 0.05	-0.02 ± 0.001	-0.02 ± 0.001
F765ϕ	0.69 ± 0.2	27 ± 1.9	40 ± 9	1.3 ± 0.3	-0.11 ± 0.02	-0.08 ± 0.03
			**Double mutant cycles**			
	k_{on} (μM^{-1}s^{-1})	k_{off} (s^{-1})	K_d (μM)	ΔΔΔG_{eq} (kcal mol^{-1})	ΔΔΔG_{TS} (kcal mol^{-1})	
G756γ/ A461α	0.43 ± 0.03	12 ± 0.6	27 ± 3	-0.55 ± 0.07	-0.08 ± 0.01	
N759η/ Y459ψ	0.57 ± 0.06	40 ± 1.0	69 ± 8	-0.52 ± 0.07	-0.11 ± 0.01	

PARM-pWT (364-570; R455K, C483A, C501A, Y524W, C566A). See Methods section in main text for errors.

Supporting References

(1) Ghose, A.K. & Crippen, G.M., *J. Chem. Inf. Comput. Sci.* **1987**, 27, 21-35.

(2) a) Dogan, J., Mu, X., Engstrom, A. & Jemth, P., *Sci. Rep.* **2013**, 3, 2076; b) Iesmantavicius, V., Dogan, J., Jemth, P., Teilum, K. & Kjaergaard, M., *Angew. Chem. Int. Ed. Engl.* **2014**, 53, 1548-1551.

(3) Giri, R., Morrone, A., Toto, A., Brunori, M. & Gianni, S., *Proc. Natl. Acad. Sci. U. S. A.* **2013**, 110, 14942-14947.

(4) Lindstrom, I. & Dogan, J., *Biochem.* **2017**, 56, 4145-4153.

(5) Dahal, L., Kwan, T.O.C., Shammas, S.L. & Clarke, J., *Biophys. J.* **2017**, 113, 2713-2722.
